# Tissue-specific regulation of PNPLA3 promotes lipid remodeling in response to dietary and temperature stress

**DOI:** 10.1101/2025.10.27.684800

**Published:** 2025-10-27

**Authors:** Panyun Wu, Yang Wang, Jonathan C. Cohen, Helen H Hobbs

**Affiliations:** 1 Department of Molecular Genetics, University of Texas Southwestern Medical Center (UTSW), Dallas, TX 75390-9046, USA; 2 Center for Human Nutrition, UTSW, Dallas, TX 75390, USA; 3 Howard Hughes Medical Institute, UTSW, Dallas, TX 75390, USA.

**Keywords:** adipose tissue, cold exposure, lipolysis, lipid droplets

## Abstract

A variant in PNPLA3, an enzyme that promotes transfer of long-chain polyunsaturated fatty acids (LCPUFAs) from triglycerides to phospholipids in lipid droplets (LDs), is a major risk factor for steatotic liver disease. At thermoneutrality, PNPLA3 is easily detected in liver and increases with feeding; the protein is undetectable in adipose tissue, despite significantly higher *Pnpla3* mRNA levels. Cold exposure or β3-adrenergic stimulation did not alter hepatic PNPLA3 but triggered a >19-fold increase in adipose PNPLA3, accompanied by a >75% reduction in *Pnpla3* mRNA. This effect occurred in cultured adipocytes and was blocked by inhibiting Protein Kinase A or AKT. The predominant cause of the kinase-dependent increase in adipocyte PNPLA3 was reduced degradation. We speculate that the tissue-specific regulation of PNPLA3 redistributes LCPUFAs to the LD surface to enhance lipid remodeling and promote fatty acid mobilization in response to feeding in liver and to cold in adipose tissue.

## Introduction

A missense variant (I148M) in patatin-like phospholipase domain-containing protein 3 (PNPLA3) is recognized as the strongest genetic risk factor for steatotic liver disease (SLD)^1^. The 148M variant is associated with increased hepatic triglyceride (TG) content and progressive liver disease due to metabolic dysfunction-associated steatotic liver disease (MASLD) or alcohol-related SLD (ALD)^1,2^. Although the contribution of PNPLA3(148M) to SLD has been intensively investigated, the physiological role of PNPLA3 remains poorly defined.

PNPLA3 belongs to a family of serine hydrolases that share a domain resembling patatin, a nonspecific acyl hydrolase widely expressed in plants^3^. In mammals, PNPLA3 shares significant sequence similarity with PNPLA2 (also known as adipose triglyceride lipase, ATGL), the principal TG lipase in most tissues^4^. Recombinant PNPLA3 exhibits TG hydrolase activity *in vitro* that is lower than that of ATGL^5,6^. Both enzymes are stimulated by α/β hydrolase domain containing protein 5 (ABHD5)^6–8^. Work from our laboratory^9^ and others^10^ has established that PNPLA3 is much more active against long chain polyunsaturated fatty acids (LCPUFAs) than monounsaturated or saturated fatty acids (FAs) of TG. In hepatocytes, PNPLA3 promotes transfer of LCPUFAs from TG and cholesteryl esters to the phospholipid (PL) monolayer surrounding the neutral lipids of lipid droplets (LDs)^9^.

While the biochemical activity of PNPLA3 has been characterized in detail, its physiological role is still not established. Early studies of the tissue-specific localization and regulation of PNLA3 showed that the enzyme is expressed most highly in liver and adipose tissue^11^. In liver, PNPLA3 expression is highly regulated by food intake, predominantly at the transcriptional level^12^. Fasting is associated with very low levels of both *Pnpla3* mRNA and protein levels in the liver. Feeding induces rapid increases in hepatic *Pnpla3* mRNA and protein that can be blocked by inactivation of the transcription factor sterol regulatory element-binding protein-1c (SREBP-1c)^12^. In liver, PNPLA3 is also subject to post-translational regulation^12^. PNPLA3 is synthesized on cytoplasmic ribosomes, but rapidly localizes to LDs, where it is stabilized^12^. The protein is degraded by the ubiquitin proteasome system (UPS)^13,14^.

Much less is known about PNPLA3 expression in adipose tissue, in part because of the dearth of antibodies that detect mouse PNPLA3. As a consequence, studies have focused on the *Pnpla3* mRNA without analyzing the PNPLA3 protein. In mice, *Pnpla3* mRNA levels are significantly higher in adipose tissue than liver, even in the fed state^11,12^, and increase in response to nutritional and hormonal signals, such as refeeding and thyroid hormone^12,15^. Cold exposure or β-adrenergic stimulation decrease *Pnpla3* mRNA in the adipose tissue of mice, as well as in cultured adipocytes^16^. Although *Pnpla3* mRNA levels increase during adipocyte differentiation^11^, the enzyme is not required for adipose tissue formation in mice^17,18^.

To further define the metabolic role of PNPLA3, we compared *Pnpla3* mRNA and protein levels in various fat depots to those in mouse liver. While levels of *Pnpla3* mRNA were up to 8-fold higher in the various adipose tissue depots than liver, PNPLA3 protein was undetectable by immunoblotting using either cell lysates or isolated LDs. Interestingly, cold exposure markedly increased PNPLA3 protein levels in adipose tissue, especially brown adipose tissue (BAT), despite an associated decrease in *Pnpla3* mRNA levels. Here we show that PNPLA3 levels in BAT are predominantly regulated at the post-transcriptional level by β-adrenergic receptor (βAR) signaling, rather than by SREBP-1c mediated transcriptional regulation, as occurs in the liver^12^.

The striking differences in regulation of PNPLA3 in liver and adipose tissue inform the roles of the enzyme in lipid metabolism in the two tissues. We speculate that food intake and cold exposure increase levels of PNPLA3 in liver and adipose tissue, respectively, to enhance transfer of LCPUFAs from the neutral lipids (TG and cholesteryl esters) in the core of the LD to its surface PL monolayer. These changes would be predicted to enhance the flexibility of the LD surface and promote mobilization of fatty acids.

## Results

### Dissociation between levels of *Pnpla3* mRNA and protein in adipose tissue

**Fig.1 |:**
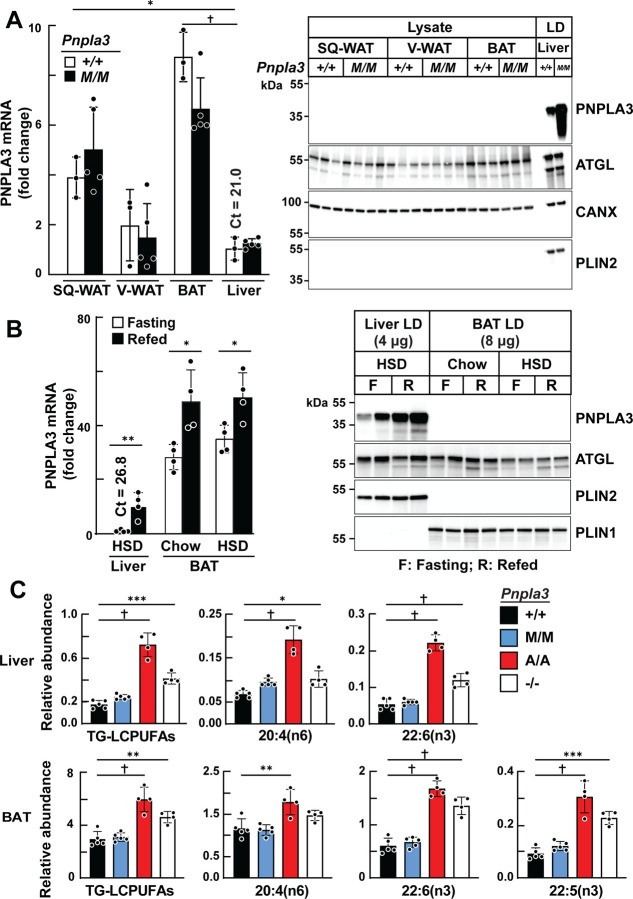
Dissociation between *Pnpla3* mRNA and protein levels in mouse adipose tissue. (A) RT-qPCR analysis of *Pnpla3* mRNA levels in subcutaneous white adipose tissue (SQ-WAT), visceral white adipose tissue (V-WAT), brown adipose tissue (BAT) and liver (left). Immunoblot analysis of PNPLA3 in adipose tissue lysates (26 μg) and liver lipid droplets (LDs) (6 μg) (right). WT and *Pnpla3*^*M/M*^ male mice (n = 3–5/group) were maintained at 30°C on a high sucrose diet (HSD) for 4 weeks. (B) WT male mice (n = 4/group) were fasted for 12 (F) or refed for 12 h (R) on regular chow or HSD. *Pnpla3* mRNA and protein were quantified as described in panel A except that LDs were isolated from liver and BAT. (C) Distribution of long chain polyunsaturated fatty acids (LCPUFAs) in BAT and liver of genetically modified mice. *Pnpla3*^*−/−*^, *Pnpla3*^*M/M*^, *Pnpla3*^*A/A*^ and WT mice (n = 4–5/group) were maintained as described for Fig.1A. Lipids from liver and adipose tissue were extracted and neutral lipids analyzed by GC-MS with internal standards. Data represent mean ± SD. (A, B) Mean values for *Pnpla3* mRNA were compared in WT and *Pnpla3*^*M/M*^, fasting and refed using Student’s *t*-test. (C) P values were determined by one-way ANOVA followed by Tukey’s multiple comparisons test; *P < 0.05; **P < 0.01; ***P < 0.001; †P < 0.0001. Ct = Cycle threshold.

We compared levels of *Pnpla3* mRNA and protein in BAT and in subcutaneous and visceral WAT (SQ-WAT and V-WAT, respectively) to those in livers in wildtype (WT) mice and in mice expressing PNPLA3(148M) (*Pnpla3*^*M/M*^ mice)^19^. Mice were maintained at thermoneutrality (30°C) for 4 weeks and fed a high-sucrose diet (HSD) to induce PNPLA3 expression^12^. Levels of *Pnpla3* mRNA were significantly higher in adipose tissue compared to liver (Fig. 1A, left). The highest level of *Pnpla3* mRNA was in BAT, followed by SQ-WAT and V-WAT. Similar results were reported previously in mice maintained at room temperature^11^. No differences in *Pnpla3* mRNA levels were seen between WT and *Pnpla3*^*M/M*^ mice. Despite the higher levels of *Pnpla3* mRNA in adipose tissue, PNPLA3 was undetectable in adipose tissue lysates when probed with our rabbit monoclonal antibody (19A6)^13^, which recognizes PNPLA3 in hepatic LDs (Fig. 1A, right). Consistent with prior findings, levels of PNPLA3 in hepatic LDs were higher in *Pnpla3*^*M/M*^ mice than in WT animals^13^.

To determine if our inability to detect PNPLA3 in adipose tissue by immunoblotting was because we used tissue lysates rather than LDs, we repeated the experiment using LDs from BAT. In liver, levels of *Pnpla3* mRNA were low after a 12-h fast and increased 11-fold with refeeding, as found previously^14^ (Fig. 1B, left). In this experiment, *Pnpla3* mRNA levels were 36-fold higher in BAT of fasted mice and increased just 1.5-fold with refeeding. Despite BAT having much higher *Pnpla3* mRNA levels, PNPLA3 protein remained undetectable in LDs from BAT (Fig. 1B, right). Other LD proteins, such as ATGL and perilipin 1 (PLIN1), were readily detected in the BAT samples, confirming that the proteins were not degraded in the samples.

### Fatty acid profiles of BAT resemble those of liver in genetically modified mice

We used an orthogonal assay to determine if PNPLA3 is expressed in adipose tissue. Previously, we showed that WT and *Pnpla3*^*M/M*^ have lower levels of LCPUFAs in TG when compared to mice expressing no enzyme (*Pnpla3*^*−/−*^ mice) or mice in which the enzyme was inactivated by substituting an alanine for the catalytic serine at residue 47 (*Pnpla3*^*A/A*^)^9^. If PNPLA3 is expressed in adipose tissue, we would expect that the pattern of enrichment of LCPUFAs in the adipose tissues be similar to that seen in liver in the *Pnpla3*^*M/M*^, *Pnpla3*^*A/A*^*, Pnpla3*^*−/−*^ and WT mice.

As observed previously, the LCPUFAs content of the TG in livers of *Pnpla3*^*M/M*^ and WT mice were depleted, whereas the LCPUFAs of the TG in the livers of *Pnpla3*^*A/A*^ and *Pnpla3*^*−/−*^ mice were enriched (Fig. 1C, top). A similar genotype-specific difference in the pattern of TG-LCPUFAs levels was found in BAT (Fig. 1C, bottom), V-WAT and SQ-WAT (Fig. S1A). No significant differences in levels of other TG-FAs in liver and BAT were detected among the groups (Fig. S1B). In liver LDs, PL-LCPUFAs levels are reciprocally related to the levels of LCPUFAs in TG, as we reported previously^9^. Therefore, we attempted to measure the PL-LCPUFA profiles of LDs isolated from BAT. Unfortunately, LD purification from BAT consistently resulted in mitochondrial contamination, a problem encountered previously by others^20^.

The genotype-specific differences in TG-LCPUFAs levels suggested that PNPLA3 is expressed in BAT despite our inability to detect it by immunoblotting (Fig. 1). To determine if the differences in FA composition in BAT were driven by PNPLA3 activity in liver, we overexpressed PNPLA3 in livers of *Pnpla3*^*−/−*^ mice and measured TG-FA composition of BAT. *Pnpla3*^*−/−*^ mice were infected with an adenovirus (Ad) expressing PNPLA3 (Ad-PNPLA3). Three days after infection, PNPLA3 was readily detected in liver, but not in BAT (Fig. S2A). The abundance of TG-LCPUFAs in BAT and liver tissues of *Pnpla3*^*−/−*^ mice was approximately double that of WT mice at baseline (Fig. S2B). Liver-specific PNPLA3 overexpression normalized levels of hepatic TG-LCPUFAs (Fig. S2B), but had no effect on TG-LCPUFAs levels in BAT (Fig. S2B) or V-WAT (Fig. S2B). No significant differences in other FA levels were seen in TGs from the liver or BAT of these mice (Fig. S2C).

We concluded that PNPLA3 is indeed expressed in BAT, albeit at levels below those that could be detected with our antibody by immunoblotting. Alternatively, it is possible that PNPLA3 is expressed intermittently in BAT, and not when we sampled the tissues.

### Cold exposure increases PNPLA3 while decreasing levels of *Pnpla3* mRNA in adipose tissue

**Fig.2 |:**
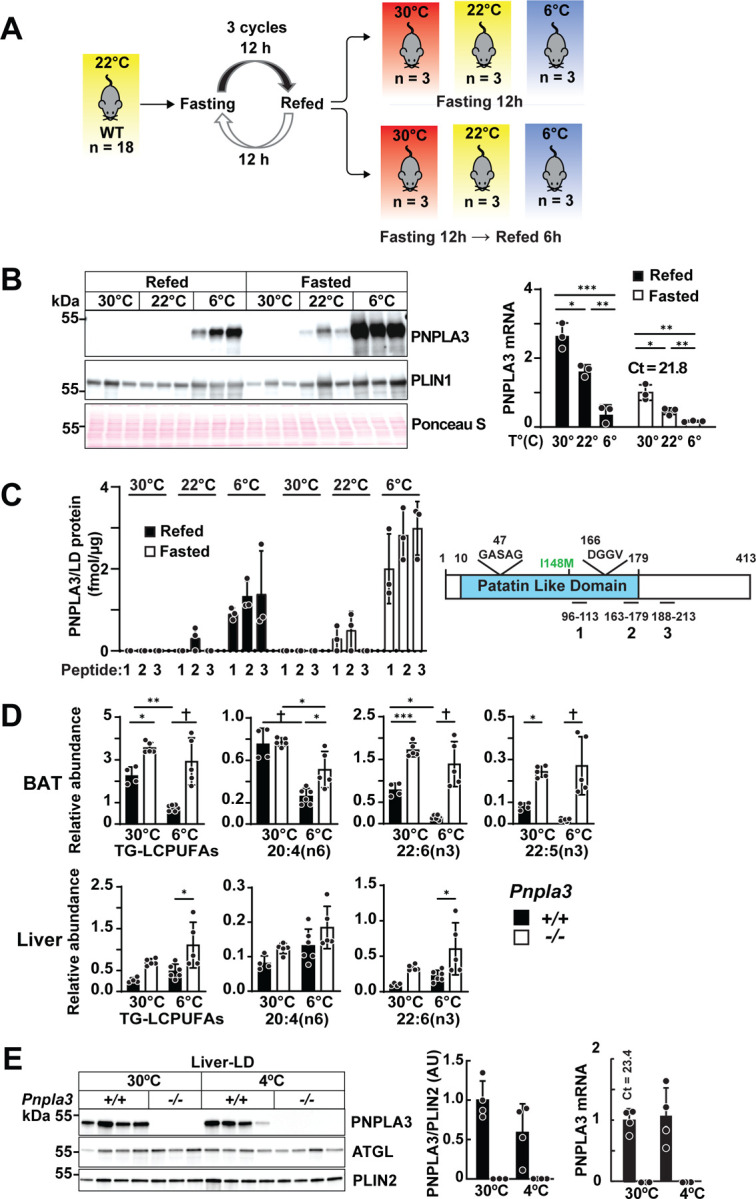
Cold exposure stimulates PNPLA3 protein expression and decreases *Pnpla3* mRNA in BAT. (A) Schematic diagram of the experimental protocol. (B) Immunoblot (left) and RT-qPCR (right) analysis of PNPLA3 protein and *Pnpla3* mRNA in BAT of WT mice maintained at 6°C, 22°C or 30°C for 12 h, and killed under fasted or refed status. (C) Quantification of PNPLA3 in BAT by selected reaction monitoring (SRM) in mice described in panel B (left). Three PNPLA3-specific, isotope-labeled peptides (96–113, 163–179, and 188–213) used as internal standards are illustrated on the right. (D) Relative abundance of TG-LCPUFAs in the BAT (upper) and liver (lower) from WT and *Pnpla3*^***−/−***^ mice (n = 4–6/group) maintained at 30°C or 6°C for one week. (E) Immunoblot analysis of PNPLA3 in hepatic lipid droplets (LDs) from mice (n = 3–4/group) maintained at thermoneutrality (30°C) or in cold (4°C) for 12 h with free access to food. Data represent mean ± SD. Groups were compared using one-way ANOVA followed by Tukey’s multiple comparisons test. *P < 0.05; **P < 0.01; ***P < 0.001; †P < 0.0001.

We then examined the effect of cold exposure on PNPLA3 expression in BAT since it had the greatest abundance of *Pnpla3* mRNA of any tissue and plays a central role in thermogenesis^21^.Mice were maintained at room temperature (22°C) with a 12-h fasting (light)/12-h refeeding (dark) cycle (Fig. 2A). Following the final fasting–refeeding cycle, mice were divided into three groups and maintained at 3 different temperatures (30°C, 22°C, and 6°C). In each group, half the mice were fasted for 12 h, and the other half were fasted for 6 h then refed for 6 h. All animals were killed at the end of the experiment. PNPLA3 protein was undetectable in LDs from the BAT of mice maintained at thermoneutrality (30°C) (Fig. 2B). At 22°C, PNPLA3 was detected in BAT of fasted mice, but not refed mice. At 6°C, PNPLA3 appeared in BAT from both fasted and refed mice. The cold-induced increases in PNPLA3 protein levels were accompanied by marked reductions in levels of *Pnpla3* mRNA (Fig. 2B, right). Fasting also caused a dissociation between PNPLA3 protein and *Pnpla3* mRNA levels in BAT (Fig. 2B).

Targeted quantification of PNPLA3 by triple quadrupole mass spectrometry with selected reaction monitoring (SRM) was used to confirm the observed increase in PNPLA3 protein in BAT of cold-exposed animals (Fig. 2C, left). No PNPLA3 peptides were detected in BAT of mice maintained at thermoneutrality for 12 h (Fig. 2C, left). Only trace levels were detected in BAT from refed mice at 22°C (0.3 fmol PNPLA3/µg protein), while peptide levels were robustly increased in refed, and especially in fasted, mice at 6°C.

PNPLA3 levels were also increased in the white adipose tissue depots in cold-exposed mice that fasted for 12 h (Fig. S3A). Thus, in contrast to liver, PNPLA3 protein levels were increased by fasting in both BAT and WAT of cold-exposed mice.

### PNPLA3 expression in BAT is associated with a decrease in TG-LCPUFAs in mice

Given that PNPLA3 levels are inversely related to TG-LCPUFAs content in hepatic LDs^9^, we measured and compared levels of selected TG-LCPUFAs in BAT of WT and *Pnpla3*^*−/−*^ mice maintained at 30°C or 6°C for 1 week. TG-LCPUFAs levels decreased by ~69% with cold exposure in BAT of WT mice, but remained unchanged in *Pnpla3*^*−/−*^ BAT (Fig. 2D, top). No TG-LCPUFAs reductions, or changes in other FAs (C16:0, C18:1, C18:2, and C18:3) were seen in the liver of cold exposed mice (Fig. 2D, bottom and Fig. S3B). Despite the increased levels of expression of PNPLA3 in WAT (Fig. S3A), we did not see changes in the TG-LCPUFAs in the WAT depots of cold-exposed mice (Fig. S3C). We concluded that cold exposure enhances PNPLA3 expression in adipose tissue, which in BAT results in a re-distribution of LCPUFAs among lipid classes.

### PNPLA3 expression is associated with a reduction in levels of circulating free LCPUFAs

We examined the effect of 7 days of cold exposure on circulating free LCPUFAs in WT and *Pnpla3*^*−/−*^ mice (Fig. S3D). No significant increase in LCPUFAs was seen in the cold-exposed WT mice. In contrast, plasma LCPUFAs increased 4-fold in *Pnpla3*^*−/−*^ mice after cold exposure. We speculate that the cold-induced increase reflects a failure of transferring LCPUFAs from TG to PL in adipose tissue in the absence of *Pnpla3*. As a consequence, the LCPUFAs are hydrolyzed from TGs in adipose tissue and released into the circulation.

### No change in hepatic *Pnpla3* mRNA or protein levels with cold exposure

To determine if cold exposure also regulates PNPLA3 expression in liver, we measured mRNA and protein on LDs from mice maintained at thermoneutrality (30°C) or at 4°C for 12 h. No significant differences in levels of hepatic PNPLA3 protein or mRNA were detected in the cold-exposed mice (Fig. 2E). FA composition was likewise unchanged (Fig. 2D, bottom). Thus, cold exposure exerts a tissue-specific effect on PNPLA3 protein expression, selectively enhancing its expression in BAT.

### The metabolic response to cold exposure is preserved in *Pnpla3*^*−/−*^ mice

**Fig.3 |:**
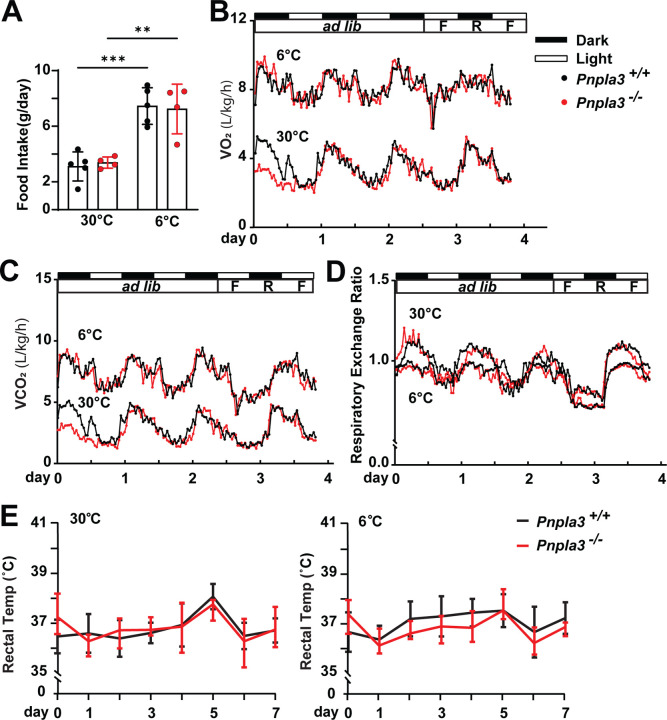
Indirect calorimetry analysis of *Pnpla3*^*−/−*^ and WT mice after cold exposure. (A–D) Effect of environmental temperature on food intake (A), VO_2_(L/kg/h; B), VCO_2_ (L/kg/h; C), respiratory exchange ratios (RER; D) in *Pnpla3*^***−/−***^ and WT mice. Mice (male, 8–10 wks, n = 4/group) were housed at 22°C on HSD for 2 weeks. Mice were then housed individually in metabolic cages for acclimatization. After two days, mice were transferred to metabolic chambers set at 6°C, 22°C and 30°C. Mice had free access to HSD during the first 60 h, followed by 12 h of restricted feeding, 12 h of refeeding, and a final 12-h fasting period. (E) Rectal temperature of WT and *Pnpla3*^***−/−***^ mice (male, n = 4–5/group) maintained at 30°C or 6°C on HSD. Data are expressed as mean ± SD. Indirect calorimetry data were analyzed using repeated-measures (mixed-effects) ANOVA to account for correlations among observations from the same animal.

To investigate the role of PNPLA3 in energy homeostasis during cold exposure, we housed WT and *Pnpla3*^*−/−*^ mice in metabolic cages at thermoneutrality (30°C) and at 6°C for 1 week. As anticipated, cold exposure increased food intake, oxygen consumption (VO_2_) and carbon dioxide production (VCO_2_) and decreased the respiratory exchange ratio (RER) with cold exposure (Fig. 3A–D). No significant differences were seen between the two genotypes. Rectal temperature measurements of WT and *Pnpla3*^*−/−*^ mice maintained at 30°C or 6°C for 1 week also revealed no significant differences (Fig. 3E). Thus, PNPLA3 deficiency did not alter energy expenditure or thermogenic response under cold conditions.

### Activation of the β3 adrenergic receptor (β3-AR) increases PNPLA3 levels in BAT

**Fig.4 |:**
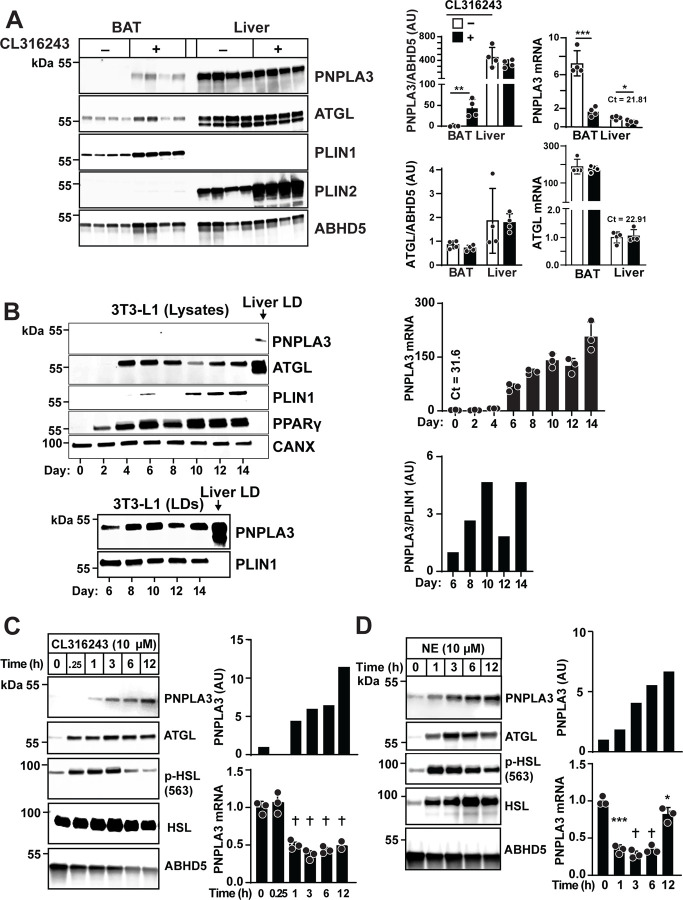
β3-AR agonists increase PNPLA3 and decrease *Pnpla3* mRNA in mouse BAT and cultured adipocytes. (A) Immunoblot (left) and RT-qPCR (right) analysis of PNPLA3 and *Pnpla3* mRNA in BAT and liver from WT mice (n = 4/group) after 6 h of treatment with CL316243 or saline. (B) PNPLA3 (upper left) and *Pnpla3* mRNA (upper right) in whole-cell lysates from differentiating 3T3-L1 cells. PNPLA3 immunoblot (lower left) and quantification (lower right) in LD fractions from the same cells. Cells were differentiated using a standard induction protocol and were collected at the specified time points after the start of induction (day 0). Hepatic LDs were used as a positive control. (C, D) Immunoblot analysis of PNPLA3 in LDs (left, upper right) and RT-qPCR analysis (lower right) of *Pnpla3* mRNA levels in 3T3-L1 cells treated with indicated β3-AR agonist: CL316243 and norepinephrine (NE). Mature adipocytes (10–12 days post-differentiation) were cultured in insulin-deprived high glucose DMEM medium for 2 days, then treated with 10 μM CL316243 (C) or 10 μM NE (D). Cells were harvested at indicated time points. Each condition was measured in triplicate plates. Data represent mean ± SD. Mean values for *Pnpla3* mRNA and protein were compared by Student’s *t*-test (A). P values were determined by one-way ANOVA followed by Dunnett’s multiple comparisons test (C, D). *P < 0.05; **P < 0.01; ***P < 0.001; †P < 0.0001.

Metabolic responses to cold exposure in BAT are mediated through β3**-**AR signaling^22^. To test if the cold-induced increase in PNPLA3 expression in BAT depends on β3-AR activation, we treated mice housed at 22°C with the selective β3-AR agonist, CL316243^23^ for 6 h. In BAT, PNPLA3 protein levels increased 43-fold (Fig. 4A, left). The dramatic increase in PNPLA3 in BAT was associated with a 76% decrease in *Pnpla3* mRNA levels (Fig. 4A, right). In contrast, PNPLA3 protein levels remained unchanged in liver while hepatic *Pnpla3* mRNA fell by 47%. ATGL mRNA and protein levels were unaffected by CL316243 treatment in either BAT or liver (Fig. 4A, bottom), indicating that the response was specific to PNPLA3 in BAT in this experiment.

We concluded that the pattern of changes in *Pnpla3* mRNA and protein expression in BAT of β3-AR-stimulated mice parallels that seen with cold exposure. To further elucidate the signaling pathway(s) responsible for the cold-induced increase in PNPLA3 levels, we established a cell culture model using murine preadipocytes (3T3-L1 cells).

### PNPLA3 expression is increased by β3-AR signaling in 3T3-L1 cells

3T3-L1 preadipocytes were differentiated into mature adipocytes following a standard protocol that included insulin supplementation^24^. During the 14-day differentiation period, *Pnpla3* mRNA levels increased ~207-fold. PNPLA3 remained undetectable in cell lysates (Fig. 4B, top). We repeated the experiment using LDs, starting at day 6 since this was the first day there were sufficient LDs to isolate. PNPLA3 was easily detected (Fig. 4B, bottom). Therefore, we monitored PNPLA3 levels in 3T3-L1 cells using LDs rather than cell lysates for all subsequent experiments.

Treatment of differentiated 3T3-L1 cells (day 12) with CL316243 induced a 4.4-fold increase in PNPLA3 protein within 1 h. PNPLA3 levels continued to increase to a level that was 11.5-fold higher than baseline at 12 h (Fig. 4C, left). Conversely, *Pnpla3* mRNA levels decreased progressively during the same period to a level that was 50% of baseline (Fig. 4C).

Similar results were obtained using norepinephrine (NE), the endogenous adrenergic ligand. PNPLA3 levels increased 1.8-fold at 1 h and 6.7-fold after 12 h (Fig. 4D, left), while *Pnpla3* mRNA levels declined progressively, reaching just 28% of baseline levels by 3 h and never returned to pretreatment levels (Fig. 4D). Thus, PNPLA3 protein and mRNA levels were also dissociated following β-adrenergic stimulation, as we had seen in the BAT of mice after cold exposure or CL316243-treatment (Fig. 2B and 4A, respectively).

### NE stimulates PNPLA3 by increasing cAMP/AKT pathway

**Fig. 5 |:**
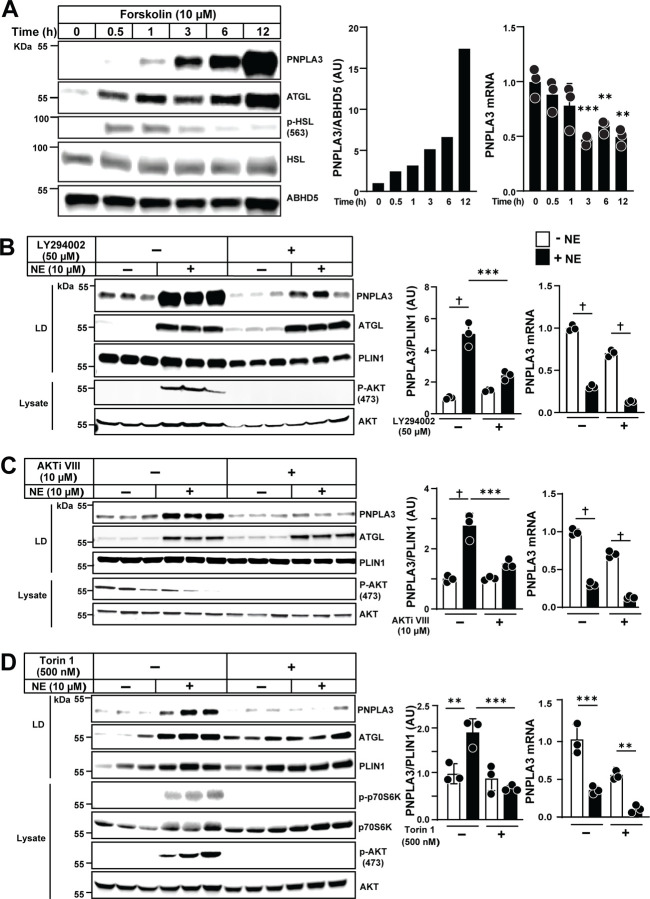
PNPLA3 expression in adipocytes is regulated by the cAMP–AKT pathway. (A) Immunoblot analysis of PNPLA3 protein levels on LDs (left, middle panels) and RT-qPCR analysis (right) of 3T3-L1 cells treated with forskolin. Mature adipocytes (10–12 days post-differentiation) were cultured in insulin-deprived high glucose DMEM medium for 2 days, then treated with 10 μM forskolin. (B–D) Western blot analysis (left, middle) of PNPLA3 on LD and relative *Pnpla3* mRNA (right) in 3T3-L1 cells treated with norepinephrine (NE) in the presence or absence of the indicated kinase inhibitors: LY294002, AKTi VIII, Torin 1. Mature adipocytes were insulin-starved for 2 d, then pretreated overnight with LY294002 (50 μM), AKTi VIII (10 μM), Torin 1 (500 nM). The next day, cells were stimulated with NE (10 μM) or vehicle (DMSO) in fresh medium containing 2% BSA, with or without the respective inhibitor, and harvested after 3 h (n = 3/group). Data represent mean ± SD. P values were determined by one-way ANOVA followed by Dunnett’s multiple comparisons test (A) and by one-way ANOVA followed by Tukey’s multiple comparisons test (B–D). *P < 0.05; **P < 0.01; ***P < 0.001; †P < 0.0001.

To determine if the NE-stimulated increase in PNPLA3 protein was mediated via the cyclic AMP (cAMP)–protein kinase A (PKA) pathway, we treated 3T3-L1 cells with forskolin, an adenylyl cyclase (AC) activator^25^. Within 30 min, phosphorylation of hormone-sensitive lipase (HSL) and an increase in ATGL were observed (Fig. 5A, left). PNPLA3 protein levels also progressively increased, first becoming detectable at 1 h (Fig. 5A). Coincident with the increase in PNPLA3 protein, levels of *Pnpla3* mRNA fell. The increase in PNPLA3 protein was blocked by co-treatment of the cells with a PKA inhibitor, H-89^26^ (Fig. S4A). Treatment with 8-Bromo-cAMP, a cAMP analog, also increased PNPLA3 levels (1.7-fold at 30 min and 8.5-fold after 6 h) while decreasing *Pnpla3* mRNA levels (Fig. S4B).

Because NE also activates phosphatidylinositol 3-kinase (PI3K), leading to AK strain transforming 1 kinase (AKT) activation^27^, we examined the effect of altering this signaling pathway on PNPLA3. Treatment with NE caused a 5-fold increase in PNPLA3 protein, an effect that was blocked by co-administration of a PI3K antagonist, LY294002^28^ (Fig. 5B) or an AKT1/2 antagonist, AKTi VIII^29^ (Fig. 5C).

To test the role of mTOR signaling, we treated 3T3-L1 adipocytes with NE in the presence or absence of Torin 1, an inhibitor of mTOR complex 1 (mTORC1) and mTOR complex 2 (mTORC2)^30^. Torin 1 blocked the NE-induced increase in PNPLA3 and further reduced *Pnpla3* mRNA levels (Fig. 5D). In contrast, rapamycin, which inhibits only mTORC1^31^, suppressed phosphorylation of S6 kinase (S6K)^32^, but did not interfere with the NE-induced increase in PNPLA3 (Fig. S4C). The finding that Torin 1, but not rapamycin, inhibited the NE-induced increase in PNPLA3 in these cells, implicated mTORC2 in the NE-induced increase in PNPLA3. As no mTORC2-specific inhibitors are available, and we failed to establish an protocol that inactivated mTORC2 using siRNAs, we cannot rule out the possibility that there is crosstalk between mTORC1 and mTORC2, and that inhibition of both pathways is required to abolish NE-induced increase in PNPLA3.

### Adrenergic signaling reduces PNPLA3 degradation

**Fig.6 |:**
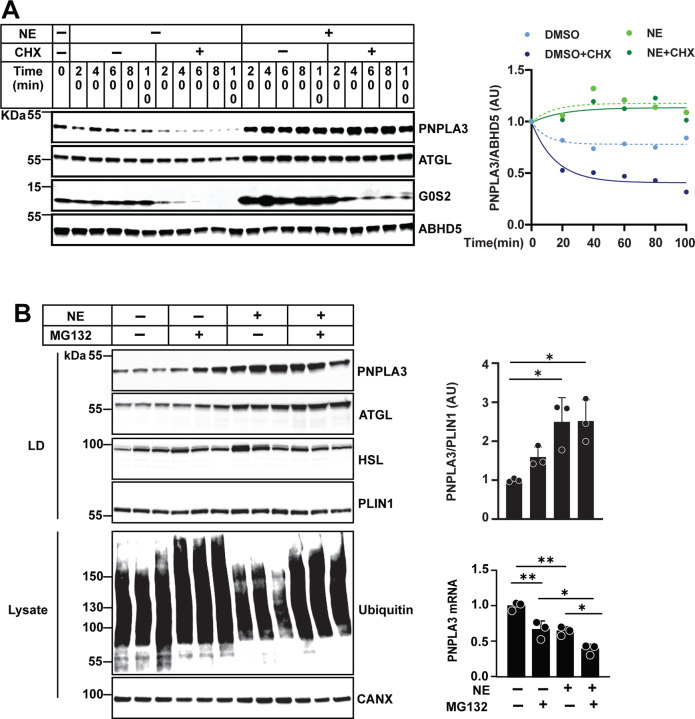
β-AR stimulation increases PNPLA3 protein levels by inhibiting UPS-mediated degradation. (A) Immunoblot analysis (left) and degradation curve (right) of PNPLA3 on LDs of 3T3-L1 cells treated with cycloheximide (CHX) and norepinephrine (NE). Cells were plated and induced to differentiate as described in Methods. On day 13, cells were treated with 10 μM NE or vehicle (DMSO) in the presence or absence of 10 μM CHX, using fresh medium containing 2% BSA. Cells were harvested at the specified time points. A degradation curve was generated by fitting nonlinear regression with a one-phase exponential decay model to individual data points. (B) Immunoblot analysis of PNPLA3 levels on LD proteins of 3T3-L1 cells treated with MG132 and NE (left and upper right panels). RT-qPCR analysis of *Pnpla3* mRNA levels (lower right). Mature 3T3-L1 adipocytes were treated with 10 μM NE or vehicle (DMSO) in the presence or absence of 10 μM MG132 for 3 h (n = 3/group). Data represent mean ± SD. P values were determined by one-way ANOVA followed by Tukey’s multiple comparisons test; *P < 0.05; **P < 0.01.

To assess whether NE increases PNPLA3 levels by reducing its degradation, we inhibited protein synthesis using cycloheximide in 3T3-L1 cells and monitored PNPLA3 levels over the ensuing 100 min^33^. In these experiments, insulin (5 μg/ml) was withdrawn on Day 0 so that baseline PNPLA3 levels were high. Under basal conditions, PNPLA3 levels remained quite stable over 100 min (Fig. 6A, left). In the presence of cycloheximide, PNPLA3 levels fell more rapidly (T1/2 = 40 min). Co-treatment with NE and cycloheximide extended the half-life of PNPLA3 (T1/2 > 100 min). These results suggested that NE stabilizes PNPLA3 by slowing its degradation, although it is also possible that the effect of cycloheximide was indirect.

We have shown previously that PNPLA3 in the liver undergoes ubiquitin-dependent proteasomal degradation^13,34^, we treated 3T3-L1 cells with NE in the absence or presence of the proteasomal inhibitor MG132^35^. Treatment with MG132 for 3 h resulted in a 1.6-fold increase in PNPLA3 (Fig. 6B). The level of PNPLA3 increased to higher level after treatment with NE. Addition of MG132 to the NE-treated cells did not cause any further increase in PNPLA3. Thus, NE stabilizes PNPLA3, likely by inhibiting its degradation via the ubiquitin-proteasome pathway. We cannot rule out the possibility that enhanced translation also contributes to the NE-induced PNPLA3 accumulation, as occurs in the integrated stress response and other adaptive pathways.

### Reduced ribosome association of *Pnpla3* mRNA in NE-treated adipocytes

We excluded several possible mechanisms that could explain the divergence between *Pnpla3* mRNA and PNPLA3 protein levels associated with cold-exposure and adrenergic stimulation in BAT, including differential splicing. We performed RNA-Seq of BAT and liver in 15-week old male mice that were maintained at 30°C or 6°C for 12 h. Seven mRNA splice variants of PNPLA3 were identified with two major forms that differed only in the noncoding regions (Fig. S5A, upper). The abundance of these transcripts (Fig. S5A, bottom) and their polyadenylation patterns (Fig. S5B) were unchanged by cold exposure.

We also ruled out the possibility of cold promoting the trafficking of PNPLA3 transcripts from the nucleus to the cytoplasm in the cold or upon adrenergic stimulation. Nuclear-cytoplasmic fractionation revealed that >80% of *Pnpla3* mRNA resided in the cytosol, independent of treatment with NE (Fig. S5C). This pattern of distribution was similar to that seen for mRNAs encoding ATGL, ABHD5, PLIN1, and HSL, whereas two mitochondrial RNAs (MT-CO3 and -ND1) and a small nuclear RNA (U6) were restricted to the cytoplasmic and nuclear compartments, respectively, as expected (Fig. S5C).

**Fig.7 |:**
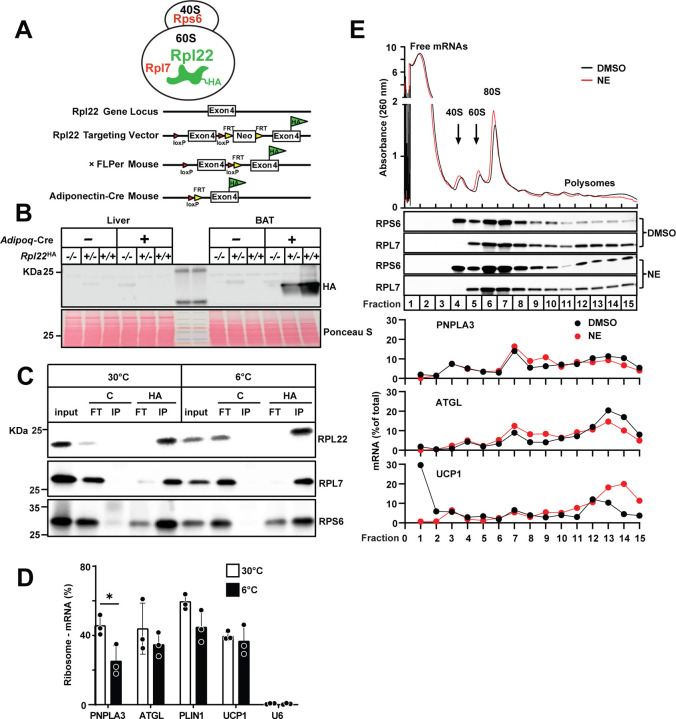
Binding efficiency of *Pnpla3* mRNA and ribosome in mouse adipose tissue and adipocytes. (A) Schematic of the generation of adipose tissue-specific RPL22-HA RiboTag mice. (B) Immunoblot of RPL22-HA in liver and BAT from adipose tissue-specific *Rpl22*^*HA*^ knock-in mice. (C) Immunoblot analysis of ribosome protein levels after immunoprecipitation of ribosomes via HA tag. (D) RT-qPCR analysis of the proportion of ribosome-associated mRNA in BAT from mice (n = 3/group) maintained at thermoneutrality (30°C) or cold (6°C) for 12 h. (E) Polysome profiling of 3T3-L1 adipocytes following norepinephrine (NE) or DMSO treatment for 3 h. Cytoplasmic lysates were separated on 10–50% sucrose gradients by ultracentrifugation. Polysome profiles were recorded by absorbance at 260 nm (upper); ribosomal subunit distribution was verified by immunoblotting for RPL7 and RPS6 (middle); target mRNAs across fractions were quantified by RT-qPCR and expressed as percentage of total (lower). Data represent mean ± SD. Statistical analysis was performed using a two-tailed Student’s *t*-test; * P < 0.05.

We then tested if NE alters translational efficiency of *Pnpla3* mRNA by modulating ribosome association with the transcript. For these experiments, we used mice in which the ribosome protein L22 (RPL22) was tagged with hemagglutinin (HA) selectively in adipocytes, as described in the Methods (Fig. 7A)^36,37^. Immunoblotting confirmed that the tagged protein was expressed in adipose tissue, but not in liver (Fig. 7B). Immunoprecipitation using an anti-HA antibody recovered intact ribosomes containing RPL22 plus two other ribosomal proteins, RPL7 and RPS6 (Fig. 7C). Quantitative reverse transcription polymerase chain reaction (RT-qPCR) revealed that ~49% of *Pnpla3* mRNA was bound to ribosomes in mice kept at 30°C, and the fraction was decreased 57% following cold exposure (Fig. 7D), whereas ribosome association of mRNAs encoding ATGL, PLIN1, and UCP1 was unchanged. Therefore, the increase in PNPLA3 protein in the BAT of cold-exposed mice could not be attributed to an increase in ribosome association with *Pnpla3* mRNA in the cold.

To test if NE promoted formation of polysome assembly on PNPLA3 transcripts to increase translation, we separated ribosome fractions from DMSO- and NE-treated cells by sucrose density gradients (10–50%) (Fig. 7E). Absorbance at 260 nm revealed well-resolved peaks for free mRNAs, 40S, 60S, 80S, and polysome peaks (Fig. 7E, top). No significant shift in distribution of ribosomal subunits or polysome peaks was observed between DMSO- and NE-treated groups, suggesting that overall translational activity was not markedly altered. To validate the identity of ribosomal complexes, gradient fractions were subjected to SDS-PAGE followed by immunoblotting for ribosomal proteins. RPS6 (small subunit) and RPL7 (large subunit) showed expected enrichment in monosome and polysome fractions. Notably, both proteins were detected in polyribosome fractions, confirming the integrity of assembled ribosomes in these fractions. RT-qPCR analysis revealed that the distribution of *Pnpla3* mRNA was unchanged by NE treatment. Nor were there any changes in the distribution of the mRNA encoding ATGL. In contrast, *Ucp1* mRNA exhibited an increased association with polysomes in response to NE stimulation, as has been reported previously^38^. Thus, NE stimulation does not appear to enhance *Pnpla3* mRNA translation by promoting formation of polyribosomes.

## Discussion

**Fig. 8 |:**
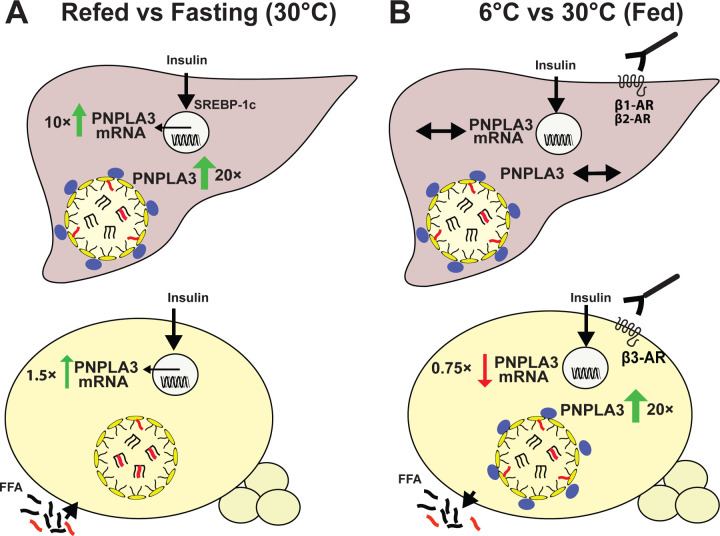
Regulation of PNPLA3 expression and lipid remodeling in liver and BAT. (A) PNPLA3 protein and *Pnpla3* mRNA levels in liver and BAT of refed (versus fasting) mice maintained at thermoneutrality. In the liver, refeeding increased *Pnpla3* mRNA and protein levels, accompanied by a redistribution of LCPUFAs from triglycerides (TGs) to phospholipids (PLs). In BAT, *Pnpla3* mRNA showed a modest increase, whereas PNPLA3 protein was not detectable by immunoblotting. LCPUFAs were predominantly present in the TGs of lipid droplets (LDs). (B) PNPLA3 protein and *Pnpla3* mRNA levels in liver and BAT of cold-exposed (6°C, fed) versus thermoneutral (fed) mice. In the liver, *Pnpla3* mRNA, PNPLA3 protein and LCPUFAs remained unchanged. In BAT, cold exposure reduced *Pnpla3* mRNA but markedly increased PNPLA3 on LDs, accompanied by a redistribution of LCPUFAs from TGs to PLs.

The major finding of this study is that expression of *Pnpla3* mRNA and protein is regulated by distinct stimuli acting through different signaling pathways in liver and adipose tissue in response to nutritional status (Fig. 8A) and thermogenic stimuli (Fig. 8B). It is well established that hepatic *Pnpla3* mRNA and protein are expressed at very low levels in fasting mice and both increase dramatically upon feeding^12^. In contrast, *Pnpla3* mRNA in BAT rose only modestly (1.5-fold) after refeeding and this increase was not accompanied by the expression of detectable PNPLA3 protein, even in LDs from BAT, the tissue depot with highest levels of *Pnpla3* mRNA (Fig. 1B). Cold exposure did not induce changes in PNPLA3 protein levels in liver (Fig. 2E). In contrast, exposure to cold exposure caused a 19-fold increase in PNPLA3 in BAT in fed mice and a >40-fold increase in fasted mice (Fig. 2B). Notably, the cold-induced increase in PNPLA3 protein in BAT occurred despite a >75% reduction in *Pnpla3* mRNA (Fig. 2B). Together, these findings reveal major tissue-specific differences in PNPLA3 expression in liver and adipose tissue in response to environmental challenges. These tissue-specific differences suggest divergent physiological roles of PNPLA3 in these two tissues.

The discordant regulation of PNPLA3 in liver and BAT in response to food intake reflects differences in the action of SREBP-1c in the two tissues. In liver, expression of PNPLA3 is primarily regulated at the transcriptional level by SREBP-1c, which binds to a sterol regulatory element (SRE) in intron 1 of the *Pnpla3* gene^12^. As a result, refeeding markedly increased PNPLA3 expression in hepatocytes. In contrast, fasting/refeeding elicited much more modest changes in *Pnpla3* mRNA in BAT (Fig. 1B). These tissue-specific differences in responsiveness of liver and adipose tissue to SREBP-1c have been reported previously^39^. Shimano and colleagues^39^ demonstrated that several lipogenic genes that strongly activated by SREBP-1c in hepatocytes do not respond to SREBP-1c in adipocytes. SREBP-1c fails to bind the SRE/E-box in chromatin immunoprecipitation assays in the fatty acid synthase (FAS) promoter in adipocytes. Moreover, deletion of SREBP-1c specifically in adipocytes has no detectable effect on TG metabolism in that tissue^39^. Thus, in adipocytes, unlike hepatocytes, increased nuclear SREBP-1c does not lead to transactivation of lipogenic genes or of PNPLA3.

We also observed discordant induction of PNPLA3 expression in liver and BAT in response to cold^40^. Cold exposure did not cause any significant changes in *Pnpla3* mRNA or protein levels in liver (Fig. 2E), but it induced an increase in PNPLA3 protein levels in BAT, despite a >75% decrease in *Pnpla3* mRNA levels (Fig. 2B). To elucidate the physiological role of cold-induced upregulation of PNPLA3 expression in BAT, we characterized the signaling pathway regulating PNPLA3 expression in adipose tissue using well-characterized agonists and inhibitors of key enzymes of major signal transduction pathways. In these studies, we focused on BAT since *Pnpla3* mRNA and protein levels were highest in this tissue (Fig. 1A). Our data indicated that the cold-induced increase in PNPLA3 protein and reduction in *Pnpla3* mRNA are transduced by β3-AR signaling through two major signaling cascades: adenylyl cyclase/cAMP/PKA and PI3K/AKT/mTORC2. Both CL316243 (a β3-AR agonist), and NE (a relatively nonselective AR agonist), reproduced the cold-induced changes in *Pnpla3* mRNA and protein expression in mouse BAT (Fig. 4A) and in cultured adipocytes (Fig. 4C and 4D). Activation of β3-AR stimulated AC to produce cAMP, which amplifies signaling by PKA activation^41^. Treatment with forskolin and 8-Bromo-cAMP, agonists of the cAMP pathway, increased PNPLA3 levels (Fig. 5A and Fig. S4B) while inducing phosphorylation of PKA, which in turn phosphorylates HSL and PLIN1, thereby catalyzing lipolysis^42,43^. Inhibition of PKA with H-89^26^ blocked NE-induced changes in PNPLA3 expression (Fig. S4A), implicating the PKA pathway in the regulation of PNPLA3 in response to cold exposure (Fig. S4D). These results implicate PNPLA3 as a downstream effector of β-adrenergic signaling, linking cold-induced lipolysis to adaptive lipid remodeling in adipose tissue.

Adrenergic signaling also activates PI3K, leading to AKT activation. Inhibition of this pathway with a PI3K antagonist (LY294002)^28^, or an AKT inhibitor (AKTi VIII)^29^, prevented the NE-induced increase in PNPLA3 protein (Fig. 5B and 5C). Downstream targets of PI3K and AKT include the mTOR complexes mTORC1 and mTORC2^44^. Treatment with Torin 1, which inhibits both mTORC1 and mTORC2, blocked the cold-induced increase in PNPLA3 (Fig. 5D), whereas rapamycin, which selectively inhibits mTORC1, did not (Fig. S4C). These results indicate that mTORC2 is required for the cold-induced increase in PNPLA3. Since inactivation of either the AKT or PKA signaling pathways prevented cold-induced increases in PNPLA3 levels (Fig. S4D), we concluded that the response of PNPLA3 to cold stress requires both pathways, perhaps acting through mTORC2, which is activated by AKT and cAMP^45^.

One of our most striking findings was the lack of correlation between levels of *Pnpla3* mRNA and protein, both at thermoneutrality and with cold exposure in adipose tissue (Fig. 1A and 2B). The discordant regulation resembles that observed for other stress-responsive proteins where precise control of protein expression is crucial. For example, the transcription factor hypoxia-inducible factor 1-alpha (HIF1A), which plays a key role in the cellular response to hypoxia. Levels of *HIF1A* mRNA respond minimally to hypoxia and yet the levels of HIF1A protein increase dramatically^46^. HIF1A is rapidly degraded in the presence of oxygen and is stabilized by hypoxia. Thus, both PNPLA3 and HIF1A are expressed at very low levels at ambient temperature and oxygen tension, and both respond to specific stressors (cold or hypoxia) with large, rapid increases in proteins that are mediated primarily by decreases in the rate of protein degradation.

For PNPLA3, the discrepancy between levels of mRNA and protein is more pronounced than in HIF1A^46^. One possibility is that cold leads to accelerated degradation of *Pnpla3* mRNA, which could be an adaptive mechanism to conserve energy^47^. Alternatively, *Pnpla3* mRNA may be sequestered in an intracellular compartment, such as stress granules, at thermoneutrality and then released and translated under cold conditions. Technical limitations prevented direct documentation of *Pnpla3* mRNA in adipose tissue stress granules. Further investigation will be required to define the potential role of stress granules in PNPLA3 regulation.

We also investigated if increased translational efficiency contributes to cold-induced increases in PNPLA3 protein. We found no evidence to support the notion that changes in mRNA splicing, polyadenylation, or nuclear export contribute to this process (Fig. S5). The hypothesis that cold exposure stimulates ribosome association and translation of *Pnpla3* mRNA was not supported: a decrease, rather than an increase, in the association of *Pnpla3* mRNA with ribosomes was found (Fig. 7D). Moreover, we failed to observe any changes in distribution of *Pnpla3* mRNA with polyribosomes, as was observed for the *Ucp1* mRNA, a protein in which translation is stimulated by cold exposure^38^(Fig. 7E). Therefore, current data do not support enhanced translation as the basis for the cold-induced increase in PNPLA3 in BAT, although we cannot exclude the possibility that ribosome stalling contributes to the high levels of *Pnpla3* mRNA in BAT of thermoneutral mice^48^.

Two lines of evidence support inhibited degradation as the major mechanism for cold-induced increases in PNPLA3 protein. First, the half-life of PNPLA3 was reduced in cycloheximide-treated adipocytes (50% decreases within 40 min, Fig. 6A). In contrast, NE treatment prolonged the half-life of PNPLA3: from 40 min to >100 min, with the addition of cycloheximide having no further effect, indicating that the major effect of NE is to block the degradation of PNPLA3. Treatment with NE produced an even greater increase in PNPLA3, which was not further enhanced by addition of MG132, suggesting the proteasome-mediated degradation of PNPLA3 was already blocked by NE. To elucidate the molecular details of how cold exposure interferes with proteasome-mediated PNPLA3 degradation will require further study.

A key unanswered question is why PNPLA3 expression is selectively increased in adipose tissue in response to cold. Does PNPLA3 contribute to cold adaptation? Generating sufficient heat to maintain body temperature is a major challenge for mice and other small mammals due to their small body size and high surface area to volume ratio, which favors dissipation, rather than retention, of heat^49^. Mice adapt to cold by uncoupling oxidative phosphorylation and allowing energy from the electron transfer chain to be released as heat^21^. Lowering ambient temperature from 30°C to 6°C doubled oxygen consumption (VO_2_), but the VO_2_ responses did not differ between WT and *Pnpla3*^*−/−*^ mice (Fig. 3). Indeed, we failed to identify any defects in cold adaptation in *Pnpla3*^*−/−*^ mice. Even after prolonged (7 days) exposure to cold temperatures, the only difference we observed between WT and *Pnpla3*^*−/−*^ mice was the retention of LCPUFAs in BAT-TG of the *Pnpla3*^*−/−*^ mice (Fig. 2D). These findings argue against a significant role for PNPLA3 in thermogenesis. It is possible that we could not detect a phenotype in *Pnpla3*^*−/−*^ mice because of adaptation. Additional studies using tissue-specific conditional inactivation of *Pnpla3* will be required to address this limitation of our studies.

The increased expression of PNPLA3 in adipose tissue in response to cold may be related to its lipid remodeling activity. During thermogenesis, sympathetic stimulation accelerates intracellular lipolysis and FA turnover, increasing FA flux in LDs. Falls in TG-LCPUFAs in the BAT of WT mice at 6°C were attenuated in *Pnpla3*^*−/−*^ mice, especially for omega 3-FAs, such as C22:6 (no change in *Pnpla3*^*−/−*^ mice vs. −80% in WT mice). The reason for the change in FA distribution among lipids in adipose tissue in the cold is currently not known. Many organisms use LCPUFAs to maintain membrane fluidity with changes in temperature^50–52^, yet PNPLA3 did not increase the LCPUFAs content of cell membranes in liver in prior studies^9^. PNPLA3 activity correlates with transfer of LCPUFAs from TG to PLs on LDs^9^. The increase in LCPUFAs in the PL monolayer may promote interactions between the LDs and other cellular organelles, such as the mitochondria. It is plausible that cold exposure stimulates expression of PNPLA3 in adipose tissue to preserve the fluidity of the PL monolayer, making the TG in LDs more accessible for lipolysis, a hypothesis that will need further testing.

## Methods:

### Mice

*Pnpla3* I148M knock-in mice (*Pnpla3*^*M/M*^) and *Pnpla3* S47A knock-in mice (*Pnpla3*^*A/A*^) were generated previously^1^. *Pnpla3*^*−/−*^ mice were a generous gift from Erin Kershaw (University of Pittsburgh, Pittsburgh, PA)^2^. RiboTag mice (*Rpl22-HA*^*fl/fl*^; strain #: 029977) were obtained from The Jackson Laboratory and crossed with mice adiponectin-Cre mice (*Adipoq*-Cre; strain#: 028020) to obtain F1 *Rpl22-HA*^*fl/+*^ Tg (*Adipoq*-Cre) offspring^3^. F1 mice were then crossed with *Rpl22-HA*^*fl/+*^mice to generate *Rpl22-HA*^*fl/f*l^ Tg (*Adipoq*-Cre) mice. Genotypes were confirmed by PCR-based genotyping using flanking oligonucleotides.

All mice were bred onto a C57BL/6J background for more than 3 generations and housed in a standard animal facility under a 12 h light/12 h dark cycle (lights on 7:00 a.m.–7:00 p.m.). Mice were fed a chow diet (PicoLab Rodent Diet 5053) *ad libitum* and maintained at 20–22°C. For dietary challenge experiments, mice were fed a high-sucrose diet (HSD; Fisher Scientific/MP Biomedicals^™^ Fat Free Diet) for 4 weeks. Prior to experiments, mice underwent dietary synchronization for 3 days consisting of 12 h fasting followed by 12 h refeeding. Unless otherwise specified, mice were euthanized after the final refeeding cycle. For temperature challenge studies, mice were housed individually in incubators maintained at either 4–6°C or 28–30°C, with free access to water and food as specified by the experimental design.

For CL316243 treatment, mice were housed at room temperature (20–22°C) and maintained on a HSD. After 3 days of dietary synchronization, mice received an intraperitoneal injection of CL316243 (1 mg/kg) at the end of the final fasting cycle, after which food was provided. Mice were killed 6 h later, and liver and brown adipose tissue (BAT) were collected and immediately placed in liquid N_2_.

A combined indirect calorimetry system (CaloSys Calorimetry System; TSE Systems, Inc., Bad Homburg, Germany) was used for all metabolic studies. Mice were acclimated to individual metabolic cages for 5 days before data acquisition. For measurements, cages were placed in temperature-controlled incubators set to 6°C, 22°C, or 30°C. Animals were monitored continuously for 96 h, consisting of 60 h with *ad libitum* access to a HSD, followed by a 12 h fasting period, a 12 h refeeding period with HSD, and a final 12 h fasting period. Oxygen consumption (VO_2_), carbon dioxide production (VCO_2_), and food intake were recorded continuously, and metabolic rate was normalized to body weight.

All animal experiments were conducted in accordance with protocols approved by the Institutional Animal Care and Use Committee of the University of Texas Southwestern Medical Center.

### Cell culture

3T3-L1 murine fibroblasts were grown to confluence in growth medium [high glucose DMEM supplemented with 10% fetal calf serum (FCS), penicillin (100 units/mL), and streptomycin (100 μg/mL)] at 37°C in 8.8% CO_2_. Two days after reaching confluence (designated as day 0), differentiation was induced by adding to the growth medium insulin (5 μg/mL), dexamethasone (1 μM), 3-isobutyl-1-methylxanthine (IBMX, 0.5 mM), and rosiglitazone (1 μM). Cells were grown for an additional 48 h^4^. Subsequently, cells were refed every other day with growth medium containing insulin (5 μg/mL). Full differentiation into mature adipocytes typically occurred within 10–12 days. Insulin was withdrawn from mature adipocytes for two days prior to the start of the experiment, unless otherwise stated.

Cells were treated with a single dose of norepinephrine (NE, 10 μM), CL316243 (10 μM), forskolin (10 μM), or 8-Bromo-cAMP (1 mM) in high glucose DMEM supplemented with 2% fatty acid–free bovine serum albumin (FAF-BSA) and harvested at the indicated time points.

For PKA inhibition experiments, cells were pretreated with H-89 (50 μM) for 1 h, followed by treatment with forskolin (5 μM) or vehicle (DMSO) in the presence or absence of H-89, using fresh medium containing 2% FAF-BSA. After 3 h of incubation, cells were harvested for analysis.

For kinase inhibitor experiments, cells were incubated overnight with LY294002 (50 μM), AKTi VIII (10 μM), Torin 1 (500 nM) or rapamycin (100 nM). The following day, cells were stimulated with NE (10 μM) or vehicle (DMSO) in fresh medium containing 2% FAF–BSA, in the presence or absence of the inhibitor. After 3 h, cells were harvested for analysis.

For proteasome inhibition experiments, cells were treated with NE (10 μM) or DMSO in the absence or presence of MG132 (10 μM) in medium containing 2% FAF–BSA for 3 h. For protein synthesis inhibition, cells were treated with cycloheximide (CHX, 10 μM) together with NE or DMSO on day 12 post-differentiation without prior insulin starvation.

### Lipid droplet isolation

Lipid droplets (LDs) from BAT were isolated as previously described^5^ with slight modifications. BAT was homogenized in 1.5 mL ice-cold Buffer A [25 mM Tricine (pH 7.6), 250 mM sucrose, and protease inhibitors] and passed through a 200-μm mesh. The homogenates were centrifuged at 2,000 *g* for 15 min at 4°C to isolate LDs. The remaining homogenates were centrifuged at 12,000 *g* for 15 min to obtain smaller LDs. The LDs were further purified by centrifugation at the same respective speeds (2,000 *g* or 12,000 *g*) for 3 min, then resuspended in 200 μL of Buffer B [20 mM HEPES (pH 7.4), 100 mM KCl, 2 mM MgCl₂, and protease inhibitors] and vortexed. Washing was repeated twice, and LD proteins were isolated by adding cold acetone followed by centrifugation at 15,000 *g* for 15 min. The protein pellet was washed with acetone/ethyl ether (50:50, v/v) solution and air-dried. The dried pellet was dissolved in PBS containing 1 M urea and 2% (v/v) SDS. After solubilization, BAT LD proteins from the same mice were pooled^6^. The same method was used to isolate fat cake from subcutaneous white adipose tissue (S-WAT) and visceral white adipose tissue (V-WAT). Hepatic LDs were isolated and purified as previously described^6^.

Adipocyte LDs were isolated as previously described with slight modifications^7^. Washed adipocytes were incubated with cold PBS and Benzonase^®^ Nuclease. Cells were disrupted by repeated pipetting and then placed on ice for 10 min. The suspended cells were transferred to a Dounce homogenizer and gently homogenized on ice using 10 strokes of a hand-held pestle. The homogenate was transferred to an Eppendorf tube and centrifuged at 1,000 *g* for 10 min at 4°C. The supernatant and floating fat layer were collected into separate tubes. One-third volume of ice-cold Buffer A containing 60% sucrose (final concentration, 20%) was added to the supernatant and gently mixed. An ultracentrifuge tube (Beckman #344059) was layered with 4.5 mL of Buffer B, followed by 5.5 ml of Buffer A carefully introduced beneath Buffer B. The cell lysate was then added to the bottom of the tube. The sample was centrifuged at 20,000 *g* for 30 min at 4°C. LDs were collected and washed three times with Buffer B. LD proteins were precipitated and solubilized using the same method described above.

### Recombinant adenoviruses generation and delivery in mice

Adenoviruses expressing either an empty vector or a fusion protein of PNPLA3 with C-terminal V5 and His tags were constructed as previously described^6^. A total of 1.5 × 10¹¹ recombinant adenoviral particles in 200 μl of saline were administered to mice via tail vein injection. Following infection, mice were subjected to three cycles of dietary synchronization and killed after the final feeding cycle.

### RNA extraction and quantitative reverse transcription polymerase chain reaction (RT-qPCR)

Total RNA was extracted from cells or tissues using an RNA extraction kit (RNeasy Plus Universal Mini Kit) according to the manufacturer’s instructions. RNA concentration and purity were determined using a NanoDrop spectrophotometer, and RNA integrity was assessed by agarose gel electrophoresis. For each sample, 1–2 μg of total RNA was reverse transcribed using random hexamer primers and reverse transcription reagents (see Table S1). RT-qPCR was performed using an Applied Biosystems real-time PCR system with SYBR Green detection (see Table S1). Each reaction was carried out in a final volume of 20 μl containing 20 ng of cDNA, 167 nM of each primer, and 10 μl of SYBR Green PCR Master Mix. Thermal cycling conditions were as follows: initial denaturation at 95°C for 10 min, followed by 40 cycles of denaturation at 95°C for 15 s, and annealing/extension at 60°C for 1 min. RNA levels were normalized to cyclophilin B or HPRT mRNA levels, and relative expression was calculated using the ΔΔCq method.

### Immunoblotting and protein quantification

Cells or tissue samples were lysed in RIPA buffer [25 mM Tris-HCl (pH 7.6), 150 mM NaCl, 1% Nonidet P-40, 1% sodium deoxycholate, and 0.1% SDS], and protein concentrations were determined using the Pierce^™^ BCA Protein Assay Kit. Samples were denatured by heating in 1× Laemmli sample buffer at 95°C for 5 min. Proteins were separated by SDS-PAGE and transferred onto nitrocellulose membranes. Membranes were incubated with primary and secondary antibodies (see Table S1), and signals were detected using SuperSignal^™^ West Pico PLUS or SuperSignal^™^ West Femto Maximum Sensitivity Substrate and imaged on Odyssey FC Imager (LI-COR). Band intensities were quantified using Image Studio Lite v5.2. A rabbit anti-mouse PNPLA3 mAb (19A6) was developed against a peptide corresponding to residues 152–309 of PNPLA3^1^.

### Triglyceride fatty acid analysis by GC-MS

Approximately 50 mg of tissue was homogenized in methanol/dichloromethane (1:2, v/v) and subjected to three-phase liquid extraction as previously described^8^. Briefly, phase separation was achieved by sequential addition of water, methyl acetate, hexane, and acetonitrile (ACN), followed by vortexing and centrifugation. The upper hexane layer (~98% TG) was collected, dried under nitrogen, and hydrolyzed in methanolic KOH containing deuterated FA standards: ²H₃₁−16:0, ²H₈−20:4, and ²H₅−22:6n3. Following extraction and derivatization with pentafluorobenzyl bromide and triethylamine in acetone, FAs were analyzed by GC-MS (Agilent 7890/5975C) using electron capture negative ionization in selected ion-monitoring mode.

Peak areas of FAs were normalized to the corresponding internal standards based on chain length: ≤C18 to ²H₃₁−16:0, C20 to ²H₈−20:4, and C22 to ²H₅−22:6n3). Data processing was performed using Mass Hunter software (Agilent).

### Quantification of PNPLA3 by Selected Reaction Monitoring (SRM)

Proteins were separated by SDS-PAGE on 4–15% gradient precast gels (Bio-Rad) and visualized by Coomassie Brilliant Blue staining. A ~10 mm gel slice (35–55 kDa) corresponding to the expected molecular weight was excised for SRM analysis. Proteins within the slice were reduced with 20 mM dithiothreitol (DTT), alkylated with 27.5 mM iodoacetamide, and digested overnight at 37°C with trypsin. Peptides were extracted from the gel and dried. The samples were reconstituted and spiked with 100 fmol of each heavy-isotope labeled peptide, corresponding to three tryptic peptides: DGLQESLPDNVHQVISGK (aa 96–113), YVDGGVSDNVPVLDAK (aa 163–179), and STNFFHVNITNLSLR (aa 188–213). Peptides were selected based on their specificity and digestion efficiency. Stable isotope-labeled standards (^13^C_6_,^15^N_2_-lysine or ^13^C_6_,^15^N_4_-arginine at the C-terminus) were synthesized by 21st Century Biochemicals at >97% purity, as determined by high-performance liquid chromatography (HPLC). Peptides were desalted using an Oasis HLB μElution plate, dried, and reconstituted in 10 μL of 2% ACN/0.1% trifluoroacetic acid in water for SRM analysis.

SRM analysis was performed on an AB Sciex 6500 QTRAP mass spectrometer interfaced with a Thermo Fisher Ultimate 3000 RSLCnano HPLC system. Spiked samples were separated on a Dionex Acclaim PepMap100 C18 reverse-phase column (75 μm × 15 cm) using the Ultimate 3000 RSLCnano HPLC system. The system was controlled by Chromeleon Xpress software (v6.8 SR10) in conjunction with Dionex Chromatography MS Link (v2.12). Peptides were separated at a flow rate of 200 nL/min using the following gradient: 0–25% B (15 min), 25–35% B (5 min), and 35–80% B (5 min). Mobile phase A consisted of 2% ACN and 0.1% formic acid in water, and mobile phase B contained 80% ACN, 10% trifluoroethanol (TFE), 10% water, and 0.1% formic acid.

Mass spectrometric analysis was carried out in positive-ion low-mass mode using a NanoSpray III source equipped with a New Objective precut 360 μ PicoTip emitter (FS360-20-10-N20–10.5CT). The source settings were as follows: curtain gas = 30, ion spray voltage = 2,450 V, ion source gas 1 = 6. Analyst Software v.1.6 was used to run the mass spectrometer, and SRM data were analyzed using Skyline v4.1.

### RNA-Seq

Total RNA was isolated using the RNeasy Plus Universal Mini Kit (Qiagen) and treated with DNase. RNA quality (RIN ≥8.5) and concentration were assessed using an Agilent TapeStation 4200 and a Qubit^®^ 4.0 Fluorometer. For library preparation, 1 μg of RNA was used with the TruSeq Stranded Total RNA LT Kit (Illumina), which includes rRNA depletion, fragmentation, cDNA synthesis, adapter ligation, and PCR amplification according to manufacturer’s instructions. Sequencing was performed on illumina NextSeq 2000 platform, generating 25–35 million reads per sample. FASTQ files were quality-checked using FastQC and FastQ_screen, mapped to the hg19 reference genome with Tophat, and duplicate reads were marked using Picard tools. Differential expression analysis was conducted with edgeR (FDR <0.05; fold change >1.5), and gene ontology enrichment was analyzed using DAVID (v6.8).

### Immunoprecipitation of polysomes

The immunoprecipitation of polysomes was performed as described previously^3^. Briefly, 100 μL of protein G magnetic beads (Dynabeads; Invitrogen) were coupled directly to 10 μL of mouse monoclonal anti-HA antibody (HA.11, ascites fluid; Covance) for 45 min in citrate–phosphate buffer (24 mM citric acid, 52 mM dibasic sodium phosphate, pH 5.0). Antibody-coupled beads were washed once in citrate–phosphate buffer (pH 5.0), and twice in immunoprecipitation buffer [50 mM Tris (pH 7.5), 100 mM KCl, 12 mM MgCl2, 1% Nonidet P-40] before being added to homogenates. BAT was weighed and homogenized using a Dounce homogenizer in polysome buffer [50 mM Tris (pH 7.5), 100 mM KCl, 12 mM MgCl2, 1% Nonidet P-40, 1 mM DTT, 200 U/mL RNasin, 100 μg/mL CHX, and protease inhibitor]. Samples were centrifuged at 10,000 *g* for 10 min to obtain a post-mitochondrial supernatant. Supernatants (400 μL) were added directly to the antibody-coupled protein G magnetic beads and rotated overnight at 4°C. The following day, samples were placed on a magnetic rack on ice and supernatants were recovered before washing the pellets three times for 5 min each in high-salt buffer [50 mM Tris (pH 7.5), 300 mM KCl, 12 mM MgCl2, 1% Nonidet P-40, 1 mM DTT, and 100 μg/mL CHX]. After washing, pellets were saved for immunoblotting and RNA extraction.

To prepare total RNA, 2.5 or 5 volumes of Qiagen RLT buffer were added to the immunoprecipitated pellets or input samples, respectively. Total RNA was isolated according to the manufacturer’s instructions using the RNeasy Mini kit (Qiagen) and quantified using a NanoDrop 1000 spectrophotometer (Thermo Scientific). RNA quality was assessed by electrophoresis on 2% agarose gels followed by ethidium bromide staining.

### Polysome profiling and RNA distribution analysis

Polysome profiling was performed as previously described^9^ with minor modifications. Briefly, cells were incubated with 100 μg/mL CHX for 10 min at 37°C to arrest ribosomes on mRNAs. Cells were then washed with ice-cold PBS containing CHX and collected by centrifugation at 500 *g* for 5 min at 4°C. Cell pellets were lysed using a Dounce homogenizer in ice-cold polysome extraction buffer [20 mM Tris (pH 7.5), 100 mM KCl, 5 mM MgCl₂, 0.5% Nonidet P-40, 100 μg/mL CHX, 1:1000 RiboLock RNase inhibitor, and protease inhibitor]. Lysates were incubated on ice for 10 min and then cleared by centrifugation at 12,000 *g* for 10 min at 4°C. The upper lipid layer was carefully removed, and the resulting cytoplasmic supernatant was used for gradient loading. Equal volumes of cytoplasmic lysate (1 mL containing 2 mg total protein) were layered onto pre-formed 10–50% (w/v) sucrose gradients prepared in gradient buffer (20 mM Tris (pH 7.5), 100 mM NaCl, 5 mM MgCl₂) using a gradient mixer. Gradients were centrifuged at 190,000 *g* (∼39,000 rpm) for 90 min at 4°C in a Beckman SW41Ti rotor. Following centrifugation, sucrose gradients were fractionated at a flow rate of 1 mL/min into 15 fractions with continuous monitoring at 260 nm using a BioComp gradient fractionation system. To assess gradient integrity and ribosomal subunit distribution, proteins from each fraction were analyzed by SDS–PAGE followed by immunoblotting for RPL7 and RPS6. RNA was extracted from each sucrose fraction using TRIzol^™^ reagent and further purified with the RNeasy Mini Kit (Qiagen) according to the manufacturer’s instructions. Purified RNA was reverse transcribed using SuperScript^™^ IV Reverse Transcriptase, and target mRNA distribution across the gradient was quantified by RT-qPCR using gene-specific primers. The relative abundance of each mRNA was calculated as the percentage of total signal across all 15 fractions.

### Subcellular localization of mRNA in nuclear and cytoplasmic fractions

Cells were collected and centrifuged at 2,000 *g* for 5 min at 4°C. The resulting cell pellet was resuspended in 300 μL of ice-cold Cell Fractionation Buffer (PARIS^™^ Kit; Thermo Fisher Scientific, AM1921) and incubated on ice for 10 min with occasional gentle mixing. Samples were then centrifuged at 500 *g* for 5 min at 4 °C to remove the lipid layer. The remaining lysate and pellet (~300 μL) were combined and thoroughly mixed. To separate cytoplasmic and nuclear fractions, samples were centrifuged at 1,000 *g* for 5 min at 4 °C. The supernatant, containing the cytoplasmic fraction, was collected. The nuclear pellet was washed by resuspending in 100 μL of Cell Fractionation Buffer, followed by centrifugation at 1,000 *g* for 3 min at 4 °C; the supernatant was discarded. RNA was extracted from both fractions, and cDNA synthesis was performed as described previously. RT-qPCR was conducted to quantify mRNA levels. The relative abundance of each mRNA in the nuclear and cytoplasmic fractions was calculated as the percentage of the total signal across both compartments.

### Poly(A) tail length analysis

The poly(A) tail length of *Pnpla3* mRNA in BAT was determined using the USB^®^ Poly(A) Tail-Length Assay Kit (Affymetrix, 76455) according to the manufacturer’s instructions, with minor modifications. Total RNA was extracted from BAT of mice maintained at either 30°C or 6°C using the RNeasy Mini Kit. For each sample, 2 μg of total RNA was subjected to guanosine/inosine (G/I) tailing in a 20 μL reaction at 37°C for 60 min, followed by termination with 2 μL of 10 × Tail Stop Solution. The tailed RNA was reverse transcribed at 44°C for 60 min using the kit-supplied RT primer and reverse transcriptase, followed by heat inactivation at 92°C for 10 min. PCR amplification was performed using a gene-specific forward primer located upstream of the *Pnpla3* polyadenylation site (5′-GCAGAAGGATTGAATGGATACA-3′), paired with either the gene-specific reverse primer (5′-TTTATTATGGACCCTTTTCCCTTA-3′) to generate products terminating before the poly(A) tail, or the universal reverse primer provided in the kit to generate products containing the poly(A) tail. Amplification products were resolved on a 5% polyacrylamide gel in 1× TBE buffer and visualized by ethidium bromide staining under UV illumination. The poly(A) tail length was calculated as the size of the poly(A) PCR product minus the distance between the gene-specific forward primer and the putative polyadenylation site.

### Quantification and statistical analysis

Data are presented as mean ± SD unless otherwise specified. Statistical comparisons between two groups were performed using a two-tailed unpaired Students’*t*-test. For multiple group comparisons, one-way ANOVA followed by Dunnett’s multiple comparison or Tukey’s multiple comparisons test was applied as appropriate. Statistical significance was defined as a *p* < 0.05. Details for statistical analyses performed for each experiment are provided in the figure legends.

## Supplementary Material

Supplement 1

## Figures and Tables

**Table 1.** Materials

Reagent	Source	Identifier
**Antibodies (Ab)**		
ABHD5	Novus	H00051099-M01
AKT	Cell Signaling Technology	9272
ATGL	Cell Signaling Technology	2138
Calnexin	Enzo Life Sciences	ADI-SPA-860-F
G0S2	Proteintech	12091-1-AP
HA	Biolegend	901513
HSL	Cell Signaling Technology	18381
P-4EBP1	Cell Signaling Technology	9451
P-AKT473	Cell Signaling Technology	4060
Phospho-(Ser/Thr) PKA	Cell Signaling Technology	9621
P-HSL563	Cell Signaling Technology	4139
PLIN1	Cell Signaling Technology	3470
PLIN2	Abcam	ab108323
P70S6K	Cell Signaling Technology	9202
P-P70S6K	Cell Signaling Technology	9205
PPARγ	Cell Signaling Technology	2435
P-ULK1	Cell Signaling Technology	5869
PNPLA3 (19A6)	Lab made	N/A
RPL22	Santa Cruz	sc-522583
RPL7	Novus Biologicals	NB100-2269
RPS6	Cell Signaling Technology	2217
Ubiquitin	Cell Signaling Technology	43124
V5	Thermo Fisher Scientific	R960-25
Peroxidase AffiniPure Goat Anti-Rabbit IgG (H+L)	Jackson Immunoresearch Laboratories	111-035-144
Peroxidase AffiniPure Donkey Anti-Mouse IgG (H+L)	Jackson Immunoresearch Laboratories	715-035-150
Rabbit TrueBlot^®^: Anti-Rabbit IgG HRP	Rockland Immunochemicals, Inc.	18-8816-33
Mouse TrueBlot^®^ ULTRA: Anti-Mouse Ig HRP	Rockland Immunochemicals, Inc.	18-8817-33

**Recombinant Viruses**		
Ad-RR5	pShuttle; Clontech	
Ad-PNPLA3 (WT)	Lab made	

**Buffers**		
4X Laemmli Sample Buffer	Bio-Rad	1610747
6X Laemmli Sample Buffer	Thermo Fisher Scientific	J61337.AC
TRIS Buffered Saline (TBS)	Sigma-Aldrich	T6664
HEPES	Thermo Fisher Scientific	15630080
PBS, pH 7.4	Gibco	10010023
RNA Gel Buffer (10X MOPS Buffer)	Fisher Scientific	50-983-261
RIPA Lysis and Extraction Buffer	Thermo Fisher Scientific	89900

**Cell Lines**		
3T3-L1 murine fibroblasts	ATCC	CL-173

**Chemicals**		
Fetal Bovine Serum (FBS)	Millipore Sigma	F0926
Bovine Serum Albumin (BSA), Cohn Fraction V	Avantor	J64944-22
cOmplete Mini EDTA-free Protease Inhibitor Cocktail	Sigma-Aldrich	11836170001
Dimethyl Sulfoxide (DMSO)	Sigma-Aldrich	D2650
Glycerol	Sigma-Aldrich	G9012
Penicillin-Streptomycin	Corning	30-002-Cl
MG132	Peptide Institute, INC	3178-v
LY294002	Selleck Chemicals	S1105
AKT inhibitor VIII	MedChemExpress	612847-09-3
Torin 1	Selleck Chemicals	S2827
Rapamycin	Sigma-Aldrich	553211
Forskolin	Millipore Sigma	F6886
8-Bromo-cAMP	Selleckchem	S7857
H-89 dihydrochloride hydrate	Millipore Sigma	B1427
Cycloheximide	Millipore Sigma	C7698
Benzonase Nuclease	Millipore Sigma	E1014-25KU
Insulin(cattle)	MedChemExpress	HY-P1156
Dexamethasone	Millipore Sigma	D4902
3-Isobutyl-1-methylxanthine	Millipore Sigma	I7018
Rosiglitazone	Millipore Sigma	R2408
Tricine	Sigma	T0377
Noradrenaline tartrate	Millipore Sigma	N1100000
CL 316,243 hydrate	Millipore Sigma	C5976
Sucrose, Ultrapure Bioreagent, J.T. Baker^™^	Fisher Scientific	02-004-331
Sodium chloride	Sigma	S9625
Potassium chloride	Millipore Sigma	P5405
Magnesium chloride hexahydrate	Millipore Sigma	M9272
Acetone, HPLC Grade, ≥ 99.5%, LabChem^™^	Fisher Scientific	LC104254
Ethyl Ether	Sigma-Aldrich	EX0185-4
Sodium dodecyl sulfate solution	Millipore Sigma	71736
Urea	Sigma-Aldrich	U5378

**Enzymes**		
Pierce^™^ Trypsin Protease, MS Grade	Thermo Scientific	90057
DNase I (Lyophilized)	Promega	Z3585
SuperScript^™^ IV Reverse Transcriptase	Thermo Fisher Scientific	18090010

**Kits**		
Pierce BCA Protein Assay Kit	Thermo Fisher Scientific	23224
PARIS^™^ Kit	Invitrogen^™^	AM1921
Power SYBR Green PCR master Mix	Applied Biosystems	4368708
TaqMan reverse transcription reagents	Applied Biosystems	N8080234
RNeasy Plus Universal Mini Kit	Qiagen	73404
SuperSignal^™^ West Pico Chemiluminescent Substrate	Thermo Fisher Scientific	34580
SuperSignal^™^ West Femto Maximum Sensitivity Substrate	Thermo Fisher Scientific	34096
TruSeq Stranded Total RNA	Illumina	20020596
Poly(A) Tail-Length Assay Kit	Invitrogen	764551KT

**Medium**		
DMEM High Glucose Medium	Corning	10-013-CV

**Oligonucleotides**		
qPCR primers:	Sequence	Supplier
*mHPRT*	CCTCATGGACTGATTATGGACAG; AATCCAGCAGGTCAGCAAAG	Integrated DNA Technologies IDT
*mCyclophilin B*	TGGAGAGCACCAAGACAGACA; TGCCGGAGTCGACAATGAT	Integrated DNA Technologies IDT
*mPNPLA3*	CGAGGCGAGCGGTACGT; TGACACCGTGATGGTGGTTT	Integrated DNA Technologies IDT
*mABHD5*	AATGTGTCCCCTGCACTTACAA; GAACATCAGCGTCCATATTCTGTT	Integrated DNA Technologies IDT
*mATGL*	GAGAGAACGTCATCATATCCCA CTT; CCACAGTACACCGGGATAAATGT	Integrated DNA Technologies IDT
*mUCP1*	ACTGCCACACCTCCAGTCATT; CTTTGCCTCACTCAGGATTGG	Integrated DNA Technologies IDT
*mPLIN1*	GGTGAGCGGGACCTGTGA; TTCTCATAGGCATTGCACACAGA	Integrated DNA Technologies IDT
*mU6*	GTGCTCGCTTCGGCAGC; AAAAATATGGAACGCTTCACGAAT	Integrated DNA Technologies IDT
*mHSL*	GGAGCACTACAAACGCAACGA; TCGGCCACCGGTAAAGAG	Integrated DNA Technologies IDT
*mCo3*	CCAAGGCCACCACACTCCTA; GGTCAGCAGCCTCCTAGATCA	Integrated DNA Technologies IDT
*mND1*	GCTTTACGAGCCGTAGCCCA; GGGTCAGGCTGGCAGAAGTAA	Integrated DNA Technologies IDT

**Software and Algorithms**		
Image Studio Lite	LI-COR	v5.2 (version)
Prism 10	GraphPad	10.2.3 (version)

**Other**		
Nitrocellulose Membrane	Bio-Rad	1620168
4-15% Criterion^™^ TGX^™^ Precast Midi Protein Gel, 26 well, 15 μl	Bio-Rad	5671085
4-15% Criterion^™^ TGX^™^ Precast Midi Protein Gel, 18 well, 30 μl	Bio-Rad	5671084
4-15% Mini-PROTEAN^®^ TGX^™^ Precast Protein Gels, 10-well, 50 μl	Bio-Rad	4561084
5% Criterion^™^ TBE Polyacrylamide Gel, 12+2 well, 45 μl 3450047	Bio-Rad	3450047
InstantBlue^®^ Coomassie Protein Stain (ISB1L)	Abcam	ab119211
Pierce^™^ Protein G Magnetic Beads	Thermo Fisher Scientific	88848
RNasin^®^ Plus Ribonuclease Inhibitor	Promega	N2615
Heavy-isotope labeled peptide-DGLQESLPDNVHQVISGK (aa 96-113)	21st Century Biochemicals	N/A
Heavy-isotope labeled peptide-YVDGGVSDNVPVLDAK (aa 163-179)	21st Century Biochemicals	N/A
Heavy-isotope labeled peptide-STNFFHVNITNLSLR (aa 188-213)	21st Century Biochemicals	N/A

## Abbreviations:

ABHD5: α/β hydrolase domain containing protein 5
AC: adenylyl cyclase
AKT: AK strain transforming 1 kinase
ALD: alcohol related SLD
ATGL: adipose triglyceride lipase
AR: adrenergic receptor
BAT: brown adipose tissue
β3-AR: β3 adrenergic receptor
cAMP: cyclic AMP
CANX: calnexin
FA: fatty acid
FAS: fatty acid synthase
GC-MS: gas chromatography-mass spectrometry
HIF1A: hypoxia-inducible factor 1-alpha
HSD: high-sucrose diet
HSL: hormone-sensitive lipase
LCPUFAs: long chain polyunsaturated fatty acids
LD: lipid droplet
MASLD: metabolic dysfunction-associated steatotic liver disease
NE: norepinephrine
PI3K: phosphatidylinositol 3-kinase
PKA: protein kinase A
PL: phospholipid
PLIN1: perilipin 1
PNPLA3: patatin-like phospholipase domain-containing protein 3
RER: respiratory exchange ratio
RT-qPCR: quantitative reverse transcription polymerase chain reaction
SLD: steatotic liver disease
SQ-WAT: subcutaneous white adipose tissue
SRE: sterol regulatory element
SREBP-1c: sterol regulatory element-binding protein-1c
SRM: selected reaction monitoring
TG: triglyceride
UPS: ubiquitin proteasome system
V-WAT: visceral white adipose tissue
