# Supplementary material for: Tissue-specific regulation of PNPLA3 promotes lipid remodeling in response to dietary and temperature stress
Source: bioRxiv. 2025 Oct 27:2025.10.27.684800. Preprint. [Version 1] doi: 10.1101/2025.10.27.684800 (PMC12636316; doi:10.1101/2025.10.27.684800)
Supplement: Supplement 1 [file NIHPP2025.10.27.684800v1-supplement-1.pdf]

bioRxiv preprint doi: https://doi.org/10.1101/2025.10.27.684800; this version posted October 27, 2025. The copyright holder for this preprint (which was not certified by peer review) is the author/funder, who has granted bioRxiv a license to display the preprint in perpetuity. It is made available under aCC-BY 4.0 International license.

Supplementary Fig.1 | Composition of fatty acids in TG from adipose tissue and liver of genetically modified mice.

(A) Relative abundance of TG-LCPUFAs in visceral white adipose tissue (V-WAT, upper) and subcutaneous white adipose tissue (SQ-WAT, lower) from *Pnpla3*$^{-/-}$, *Pnpla3*$^{M/M}$, *Pnpla3*$^{A/A}$ and WT mice. (B) Relative abundance of other fatty acids (16:0, 18:1, 18:2, 18:3) in TG from liver (upper) and BAT (lower) of the same mice. Mice (n = 4-5/group) were kept at thermoneutrality (30ºC) and fed a high sucrose diet (HSD) for 4 weeks. Data represent mean ± SD. P value were determined by one-way ANOVA with Tukey's multiple comparisons test. * P < 0.05; ** P < 0.01; *** P < 0.001; †P < 0.0001.

Supplementary Fig.2 | Composition of fatty acids in liver and adipose tissue of *Pnpla3*$^{-/-}$ and WT mice infected with vector alone (V) or Ad-PNPLA3-V5.

(A) Immunoblot of human PNPLA3 in lysates from BAT and liver of mice (n = 4-5/group) infected with either vector alone (V) or Ad-PNPLA3-V5. (B) Relative abundance of LCPUFAs in TG from liver (upper), brown adipose tissue (BAT, middle) and visceral white adipose tissue (V-WAT, lower) from the same mice. (C) Relative abundance of other fatty acids (16:0, 18:1, 18:2, 18:3) in TG from liver (upper) and BAT (lower). Data represent mean ± SD. P value were determined by one-way ANOVA with Tukey's multiple comparisons test. * P < 0.05; ** P < 0.01; *** P < 0.001; †P < 0.0001.

Supplementary Fig.3 | Composition of fatty acids in liver and adipose tissue of cold-exposed *Pnpla3*$^{-/-}$ and WT mice.

(A) Immunoblot analysis of PNPLA3 in fat cake fractions of visceral white adipose tissue (V-WAT, upper) and subcutaneous white adipose tissue (SQ-WAT, lower) from mice (n = 5/group) maintained at 30°C or 6°C for one week on a high sucrose diet (HSD). (B) Relative abundance of other fatty acids (16:0, 18:1, 18:2, 18:3) in TG of liver (upper) and BAT (lower) from WT and *Pnpla3*$^{-/-}$ mice (n = 4-6/group) maintained at 30°C or 6°C for one week on a HSD. (C) Relative abundance of LCPUFAs in TG of V-WAT (upper) and SQ-WAT (lower) from the same mice. (D) Relative abundance of free LCPUFAs in plasma from WT and *Pnpla3*$^{-/-}$ mice (n = 4/group) maintained at 30°C or 6°C for one week on a HSD, and killed after 12 h fasting. Data represent mean ± SD. P values were determined by a Student's *t*-test (A) and by one-way ANOVA with Tukey's multiple comparisons test (B-D). * P < 0.05; ** P < 0.01; *** P < 0.001; †P < 0.0001.

bioRxiv preprint doi: https://doi.org/10.1101/2025.10.27.684800; this version posted October 27, 2025. The copyright holder for this preprint (which was not certified by peer review) is the author/funder, who has granted bioRxiv a license to display the preprint in perpetuity. It is made available under aCC-BY 4.0 International license.

Supplementary Fig.4 | PNPLA3 expression in adipocytes is regulated by cAMP–AKT signaling.

(A) Immunoblot analysis (left, middle) of PNPLA3 in lipid droplets (LDs) and RT-qPCR analysis (right) of *Pnpla3* mRNA in 3T3-L1 cells treated with 5 μM forskolin +/- 50 μM H-89. (B) Immunoblot analysis of PNPLA3 in LD fractions (left, middle) and RT-qPCR of *Pnpal3* mRNA (right) in 3T3-L1 cells treated with 1 mM 8-Bromo-cAMP; cells were collected at the indicated time points. (C) Immunoblot analysis (left, middle) of PNPLA3 on LDs and RT-qPCR analysis (right) of *Pnpla3* mRNA in 3T3-L1 cells treated with 10 μM norepinephrine (NE) +/- 100 nM rapamycin. (D) Schematic of signaling pathways: CL316243 activates β3-adrenergic receptors (β3-AR), engaging the PI3K–AKT–mTORC axis (inhibited by LY294002, AKTi VIII and Torin 1) and, in parallel, the adenylyl cyclase–cAMP–PKA pathway (mimicked by forskolin or 8-Bromo-cAMP; blocked by H-89), with PNPLA3 on LDs as a downstream effector. Data represent mean ± SD (n = 2-3/group). P values were determined by one-way ANOVA followed by Tukey's multiple comparisons test (A, C) or by one-way ANOVA followed by Dunnett's multiple comparisons test (B). *P < 0.05; **P < 0.01; †P < 0.0001.

Supplementary Fig.5 | Post-transcriptional regulation of PNPLA3 expression in liver and BAT in response to temperature.

(A) RNA-seq analysis of *Pnpla3* transcripts in BAT and liver from WT mice (n = 3/group) housed at 30°C or 6°C for 12 h on a high-sucrose diet (HSD). In both BAT and liver, seven *Pnpla3* isoforms were identified (upper); Transcript-level expression (TPM) for each isoform is summarized in the bar chart (lower). (B) Polyacrylamide gel electrophoresis of poly(A)-tailed PCR products from BAT of WT mice housed at 30°C or 6°C for 12 h on HSD. (C) Partitioning of mRNAs between nuclear and cytosolic fractions of 3T3-L1 adipocytes +/- norepinephrine (NE) treatment for 6 h. Data represent mean ± SD (n = 2-3/group). P values were determined by a two-tailed Student's *t*-test; P < 0.05.

Figure S1

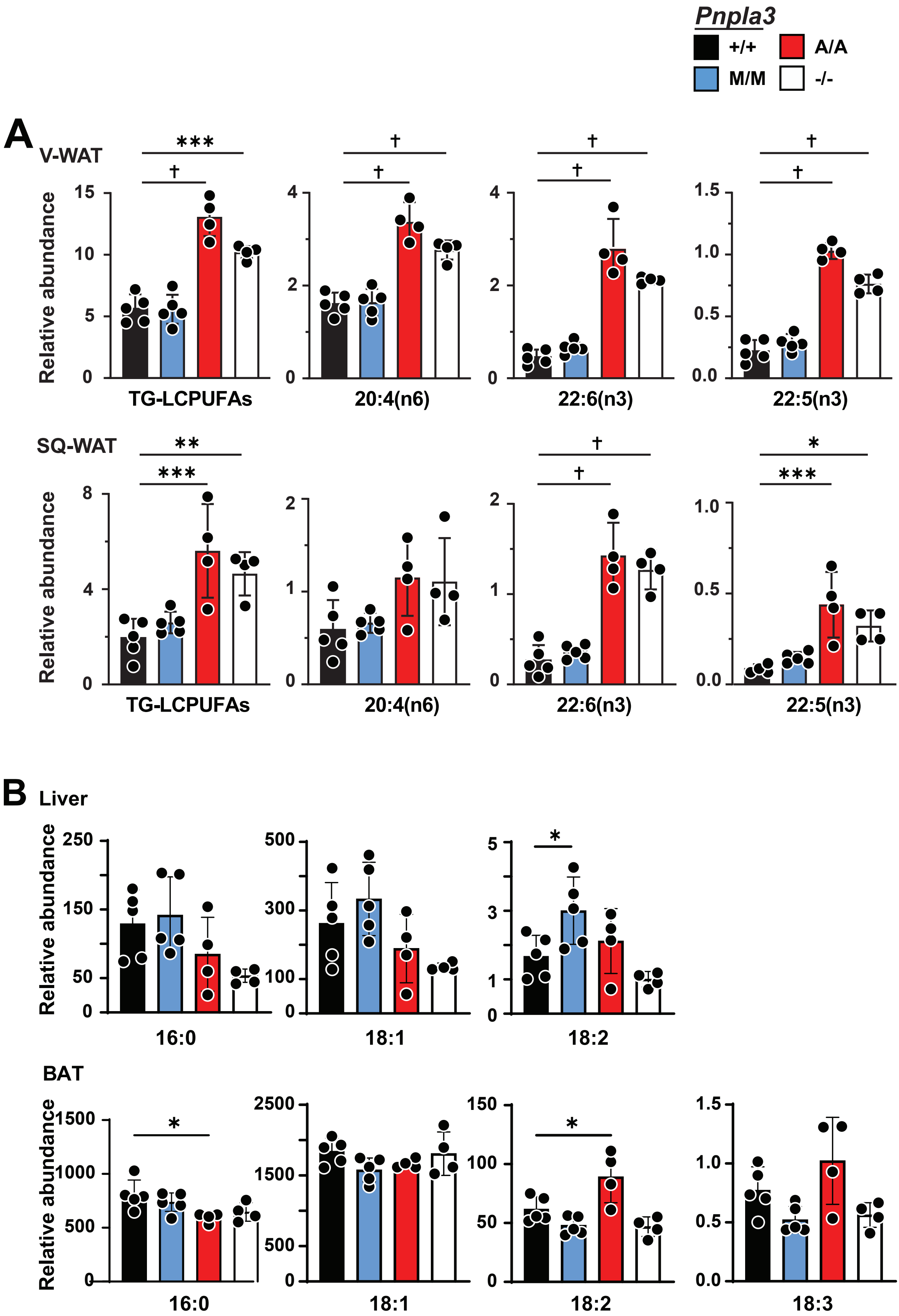

Pnpla3
+/+
A/A
M/M
-/-
A
V-WAT
Relative abundance
***
†
TG-LCPUFAs
20:4(n6)
22:6(n3)
22:5(n3)
SQ-WAT
**
*
B
Liver
16:0
18:1
18:2
BAT
18:3

Figure S2

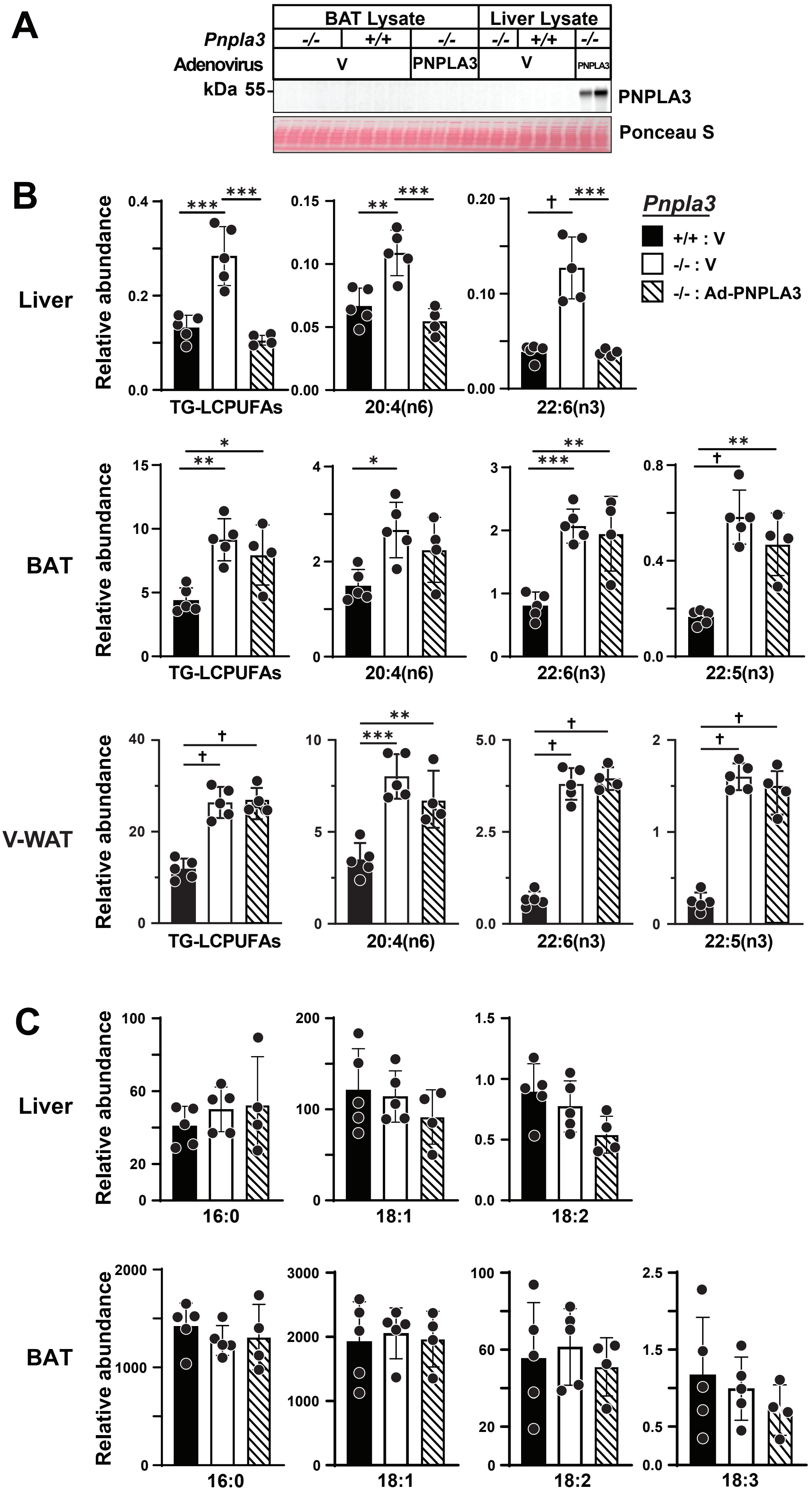

A
BAT Lysate
Liver Lysate
Pnpla3
-/-
+/+
-/-
-/-
+/+
-/-
Adenovirus
V
PNPLA3
V
PNPLA3
kDa 55
PNPLA3
Ponceau S
B
Pnpla3
+/+ : V
-/- : V
-/- : Ad-PNPLA3
Liver
BAT
V-WAT
Relative abundance
TG-LCPUFAs
20:4(n6)
22:6(n3)
22:5(n3)
C
Liver
BAT
16:0
18:1
18:2
18:3

Figure S3

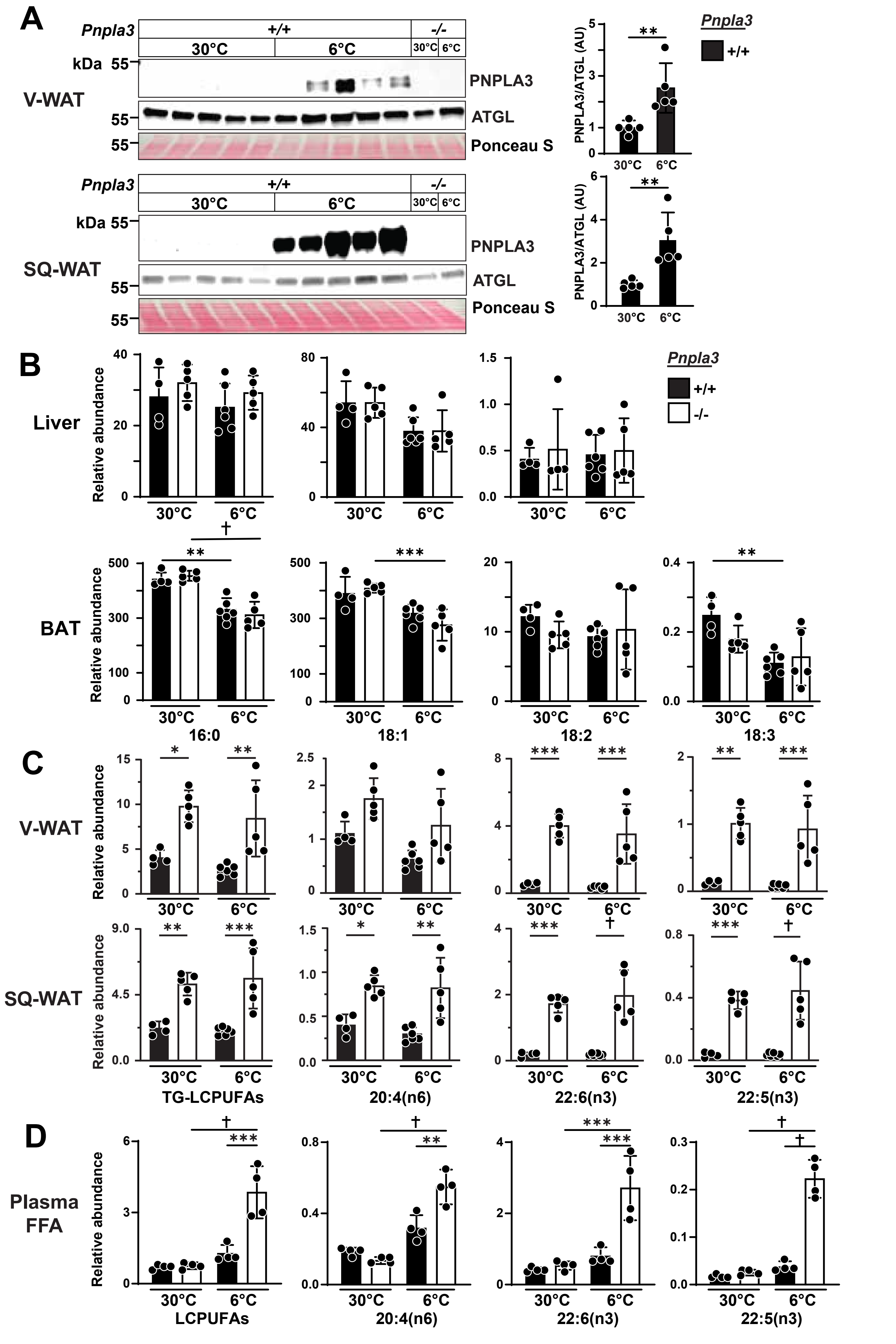

A
Pnpla3
+/+
-/-
30°C
6°C
kDa 55
V-WAT
PNPLA3
ATGL
Ponceau S
SQ-WAT
PNPLA3/ATGL (AU)
B
Liver
BAT
Relative abundance
16:0
18:1
18:2
18:3
C
V-WAT
SQ-WAT
TG-LCPUFAs
20:4(n6)
22:6(n3)
22:5(n3)
D
Plasma FFA
LCPUFAs

Figure S4

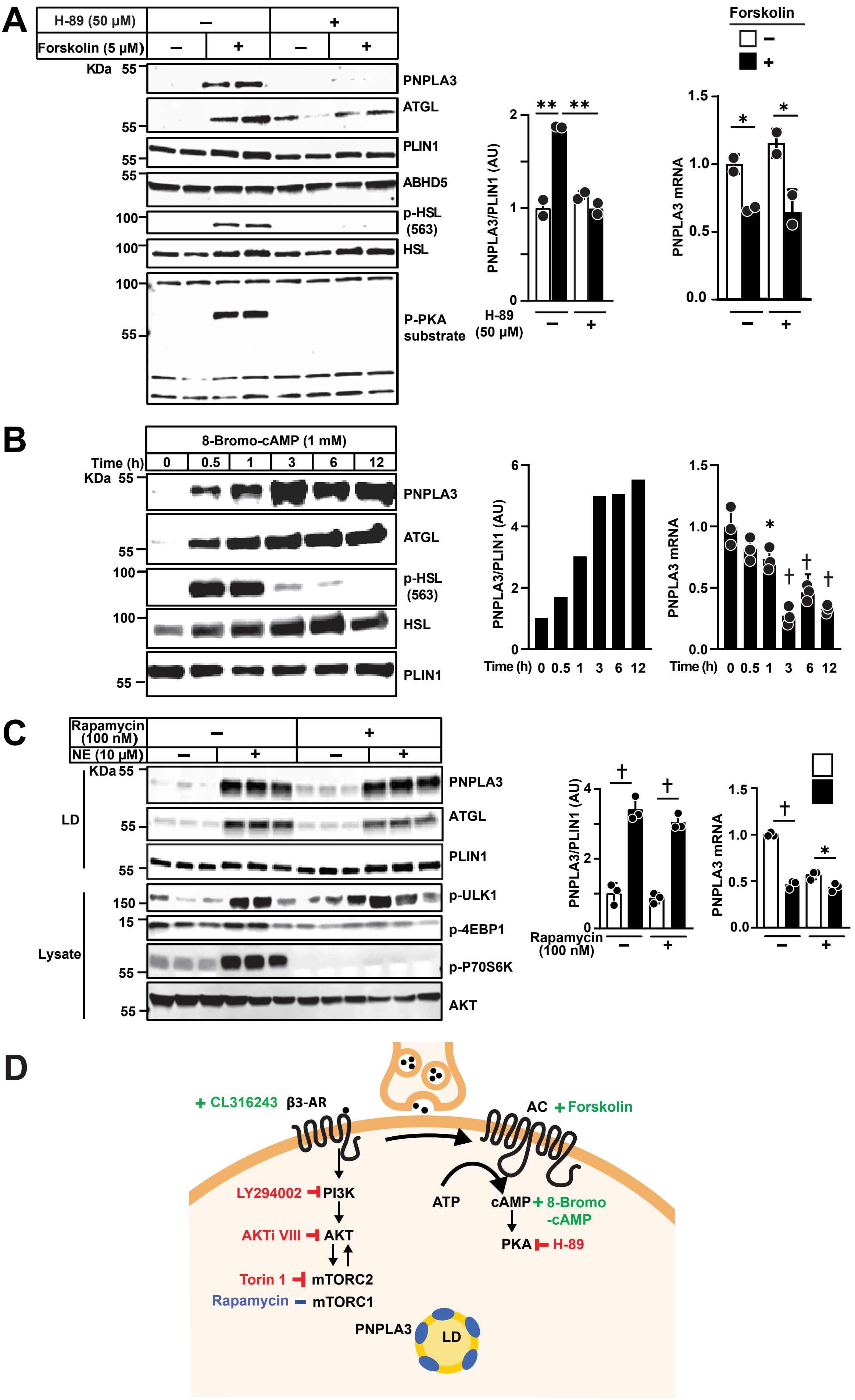
A
H-89 (50 μM)
Forskolin (5 μM)
KDa
PNPLA3
ATGL
PLIN1
ABHD5
p-HSL (563)
HSL
P-PKA substrate
PNPLA3/PLIN1 (AU)
H-89 (50 μM)
Forskolin
PNPLA3 mRNA
B
8-Bromo-cAMP (1 mM)
Time (h)
0
0.5
1
3
6
12
PNPLA3
ATGL
p-HSL (563)
HSL
PLIN1
PNPLA3/PLIN1 (AU)
PNPLA3 mRNA
C
Rapamycin (100 nM)
NE (10 μM)
LD
Lysate
PNPLA3
ATGL
PLIN1
p-ULK1
p-4EBP1
p-P70S6K
AKT
PNPLA3/PLIN1 (AU)
Rapamycin (100 nM)
PNPLA3 mRNA
D
CL316243
β3-AR
AC
Forskolin
LY294002
PI3K
AKTi VIII
AKT
Torin 1
mTORC2
Rapamycin
mTORC1
ATP
cAMP
8-Bromo -cAMP
PKA
H-89
PNPLA3
LD

Figure S5

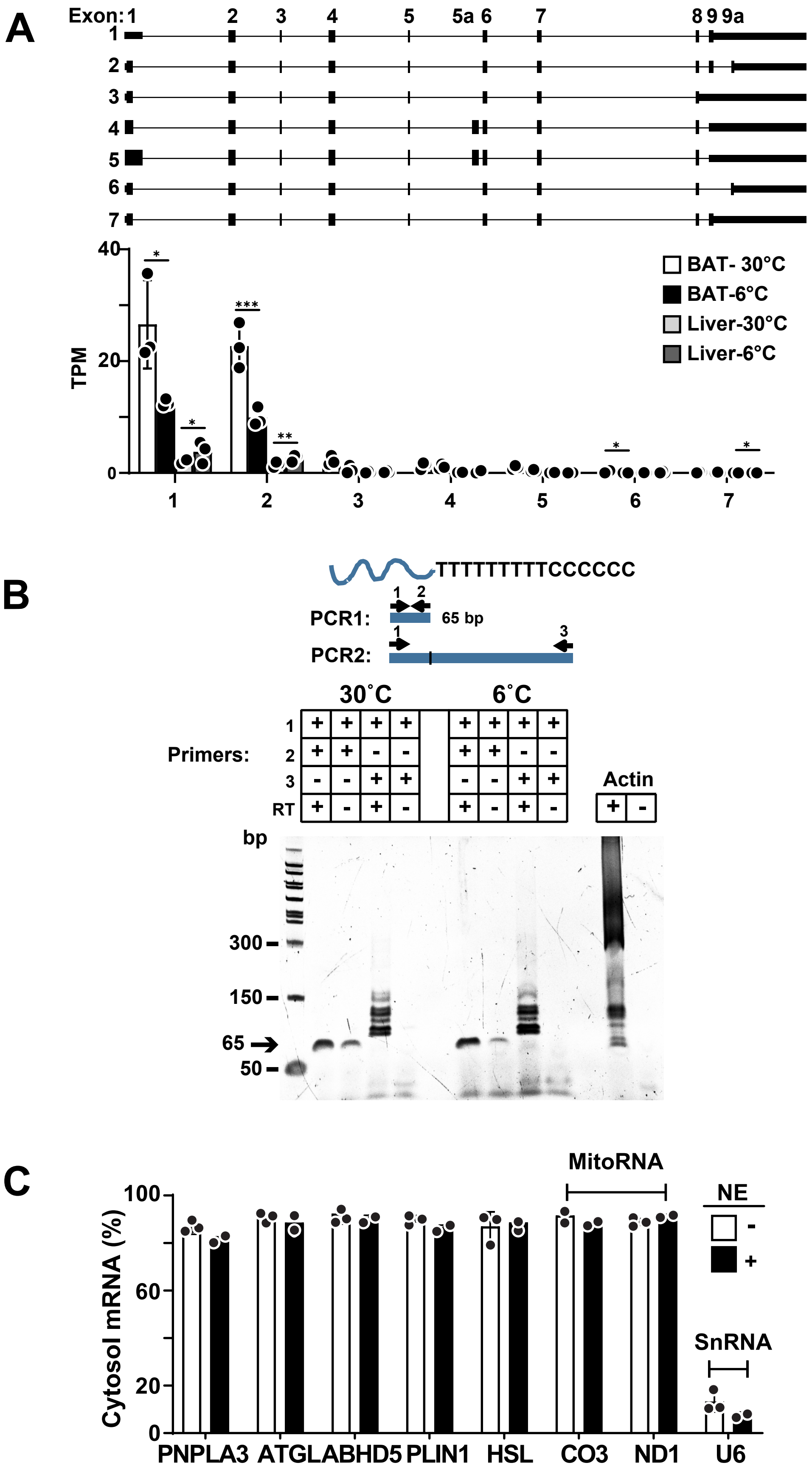

A
Exon: 1 2 3 4 5 5a 6 7 8 9 9a
TPM
BAT- 30°C
BAT-6°C
Liver-30°C
Liver-6°C
B
TTTTTTTTTCCCCCC
PCR1:
65 bp
PCR2:
30˚C
6˚C
Primers:
RT
Actin
bp
300
150
65
50
C
MitoRNA
NE
SnRNA
Cytosol mRNA (%)
PNPLA3
ATGL
ABHD5
PLIN1
HSL
CO3
ND1
U6
